# Harvesting the Spin–Orbit Interaction of Light to Generate Helicity‐Dependent Complex Rotational Motion in Optically Trapped Mesoscopic Matter

**DOI:** 10.1002/nap2.70034

**Published:** 2026-02-24

**Authors:** Ram Nandan Kumar, Jeeban Kumar Nayak, Subhasish Dutta Gupta, Nirmalya Ghosh, Ayan Banerjee

**Affiliations:** ^1^ Structured Light Laboratory, School of Physics University of the Witwatersrand Johannesburg South Africa; ^2^ Department of Physical Sciences Indian Institute of Science Education and Research Kolkata Mohanpur West Bengal India; ^3^ Tata Institute of Fundamental Research Hyderabad Telangana India

**Keywords:** birefringent liquid crystal, circularly polarized light (helicity), geometric Berry phase, optical tweezers, Pancharatnam‐Berry phase, scattering, spin angular momentum, spin‐orbit interaction‐driven optomechanics, spin‐orbit interaction of light, tight focusing

## Abstract

Spin–orbit interaction (SOI) of tightly focused light in optical tweezers underpins diverse optomechanical applications and the interconversion of spin and orbital angular momentum. Here, we demonstrate that the transfer of the spin angular momentum of a tightly focused circularly polarized beam to an on‐axis birefringent particle can indirectly generate spin in an adjacent particle, leading to exotic rotational motion reminiscent of planetary trajectories. We demonstrate simultaneous rotation and revolution of birefringent liquid crystal (LC) particles by harvesting SOI, such that its two principal governing mechanisms—the momentum‐dependent Pancharatnam–Berry (PB) phase and the anisotropy‐induced PB phase—become coupled. In our experiments, a centrally trapped LC particle in spherically aberrated optical tweezers spins under circularly polarized illumination, generating spin‐induced microfluidic flows that drive surrounding off‐axis LC particles into orbital motion. Simultaneously, interaction of the input helicity with the centrally trapped particle induces spin‐to‐spin conversion through extrinsic SOI. The helicity thereby generated indirectly then couples to the orbiting particles, imparting an additional rotation whose direction is determined by the birefringence of the central particle. A Mueller matrix model that incorporates tight focusing and scattering quantitatively explains these observations. Thus, SOI coupled with microfluidic effects establishes exotic rotational optomechanics and microswitch applications.

## Graphical abstract

Spin–orbit interaction (SOI) of light is exploited to generate helicity‐dependent complex rotational and revolution dynamics in optical tweezers. At the trap center, particle rotation is governed by the direct helicity that follows the input polarization. However, anisotropy of the centrally trapped liquid‐crystal (LC)‐induced Pancharatnam–Berry phase gives rise to extrinsic SOI, generating indirect helicities at off‐axis positions that drive simultaneous rotation and revolution, or counter‐rotation, of secondary LC particles/droplets.

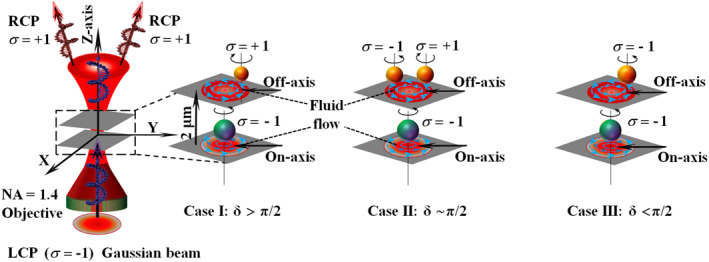

## 1 Introduction

Light is a powerful tool for manipulating matter at the microscale, enabling precise control over translation, rotation, and assembly of particles in optical trapping systems [1, 2, 3, 4, 5]. Rotational motion is typically driven by the transfer of spin angular momentum (SAM) to birefringent particles using homogeneously polarized scalar or inhomogeneously polarized vector beams [6, 7, 8, 9]. In such cases, the particles must be birefringent and therefore spin responsive. Alternatively, rotation can be induced by microstructured geometries or broken symmetries, which lead to light intensity gradients [10, 11]. Orbital motions, on the other hand, are typically controlled using beams that carry orbital angular momentum (OAM), such as Laguerre–Gauss, Bessel–Gauss, or Mathieu beams, by transferring OAM to the particles. In this case, the particles need not be birefringent in nature [2, 7, 12].

Beyond these well‐known mechanisms, the spin–orbit interaction (SOI) of light provides an additional pathway for controlling the spinning and orbiting of trapped particles [4, 9, 13]. Intrinsic SOI arises from the nonparaxial evolution of spin‐polarized fields through a momentum‐dependent Pancharatnam–Berry (PB) phase, whereas extrinsic SOI emerges from the Pancharatnam–Berry phase associated with real‐space inhomogeneous anisotropic media [14, 15, 16, 17, 18, 19]. Recent studies have demonstrated spin‐dependent wavefront engineering, beam shaping, and robust control of optical angular momentum in structured light by tailoring the PB phase in anisotropic photonic systems [20, 21]. In parallel, SOI has been extensively explored through the photonic spin Hall effect (PSHE), enabling spin‐dependent transverse shifts, vector beam sorting through PB‐phase modulation, and applications in precision metrology, sensing, and nanophotonics [22, 23, 24]. Although these studies highlight the fundamental and applied significance of PB‐phase–mediated SOI, they primarily focus on field‐level effects rather than on the optomechanical harvesting of SOI at the particle level [23, 25, 26].

An interesting question to consider is whether these two different manifestations of SOI can be combined, and what consequences such coupling might lead to. For example, in the context of particle spin, the transfer of longitudinal SAM to spin‐responsive (birefringent) materials, such as liquid‐crystal droplets, RM257, and calcium carbonate vaterite particles, is governed by the intrinsic helicity determined by the input circular polarization (RCP/LCP) of light, and is well understood as a consequence of the momentum‐dependent PB phase [5, 13, 27, 28]. In contrast, longitudinal SAM may also be indirectly harvested from SOI due to an intermediate coupling mechanism that can well involve the Pancharatnam–Berry phase. Note that, in this process, the transfer of the intrinsic helicity to a centrally trapped LC particle identifies it as the primary particle, whereas the transfer of helicity from the scattered (emerging) field of this primary particle to the surrounding LC particles identifies them as the secondary particles. Such helicity transfer has remained largely inaccessible in conventional approaches, leaving both the role and potential applications of indirect helicities generated by the harvesting of extrinsic SOI unexplored.

Here, we present a pathway for the active harvesting of SOI that extends beyond conventional PB phase modulation and PSHE studies. Specifically, we demonstrate helicity transfer arising from the combined action of intrinsic and emergent extrinsic SOI at the level of optically trapped particles. Unlike previous works that predominantly focus on helicity‐dependent transverse beam shifts or wavefront engineering at the field level, our approach enables the indirect generation and transfer of helicity mediated by a centrally trapped birefringent particle, leading to coupled spinning–orbiting dynamics of multiple microparticles. By tightly focusing a right‐ or left‐circularly polarized (RCP/LCP) Gaussian beam into a refractive‐index (RI)–stratified medium leading to spherically aberrated optical tweezers, we induce spin‐to‐spin conversion and fluid‐mediated coupling that generate simultaneous spinning and orbiting of multiple microparticles. A centrally trapped (primary) birefringent liquid‐crystal (LC) droplet rotates in response to the input (direct) helicity, generating localized spin‐driven microflows that propel surrounding off‐axis (secondary) particles into orbital motion. Simultaneously, the SOI is harvested through the interaction of the focused spin‐polarized field with the primary LC droplet producing helicity conversion through extrinsic SOI. This indirectly generated (henceforth referred to as indirect) helicity then couples to the orbiting secondary particles, imparting additional rotation whose sense is governed by the birefringence of the central (primary) LC droplet. These dynamics are thus fully governed by the transfer of helicities in a nonparaxial optical field. In our experiments, we used mesoscopic bipolar LC droplets as birefringent microparticles, which provide strong optical anisotropy and high sensitivity to SAM due to their asymmetric director configurations. In contrast, for LC droplets with a symmetric (radial) director distribution, the helicity transfer is strongly suppressed. To explain the observations, we develop a theoretical framework based on Mueller matrix formalism, which quantitatively captures the helicity‐resolved particle response. Our work thus identifies a simple yet effective route to augment SOI‐driven optomechanics by harvesting SOI and imparting fully controllable rotational dynamics to optically confined mesoscopic particles.

## 2 Theory

Tight focusing through objective lenses with large numerical apertures (NA = 1.4) leads to nonparaxial fields. To determine the electric/magnetic fields of a circularly polarized Gaussian beam under these nonparaxial conditions, we employ the angular spectrum method or the vector diffraction theory of Richards and Wolf [29, 30, 31] (detailed calculations are provided in Supporting Information S1). The electric field components (Ex, Ey, and Ez) of a focused circularly polarized (RCP/LCP) Gaussian beam in the focal plane, expressed in Cartesian coordinates (*x*, *y*, and *z*), can be described as follows:

(1) $${\left[\begin{array}{c} E_{x}^{0}\\ E_{y}^{0}\\ E_{z}^{0} \end{array}\right]}_{RCP/LCP}^{\mathrm{Gauss}}=\left[\begin{array}{c} I_{00}+I_{02}\;\cos\;2\;\psi \pm iI_{02}\;\sin\;2\;\psi \\ I_{02}\;\sin\;2\;\psi \pm i\left(I_{00}-I_{02}\;\cos\;2\;\psi \right)\\ -2iI_{01}\;\cos\;\psi \pm 2I_{01}\;\sin\;\psi \end{array}\right]$$

where $E^{0}$ denotes the output electric fields, with the suffix Gauss indicating the Gaussian beam. The terms $I_{00}$, $I_{01}$, and $I_{02}$ represent the Debye–Wolf integrals (the detailed expression is provided in Supporting Information S1) [29, 32], and ψ is the azimuthal angle in the cylindrical (or spherical) coordinate system. It is important to note that a circularly polarized Gaussian beam does not carry orbital angular momentum (OAM) but does possess spin angular momentum (SAM) with a magnitude of ±ℏ per photon, so the total angular momentum equals the SAM before focusing. However, after the tight focusing of the RCP/LCP Gaussian beam, opposite (or orthogonal) helicity and longitudinal components emerge, along with the corresponding OAM modes in the output electric field, as a consequence of SOI [14]. We now decompose the output electric fields given in Equation (1) for an input RCP or LCP Gaussian beam into their respective SAM and OAM components as follows:

(2) $$\begin{array}{c} \left[\begin{array}{c} E_{x}^{0}\\ E_{y}^{0}\\ E_{z}^{0} \end{array}\right]=I_{00}[\begin{array}{c} 1\\ \pm i\\ 0 \end{array}]+I_{02}\;\exp(\pm 2i\psi )[\begin{array}{c} 1\\ \pm i\\ 0 \end{array}]-2iI_{01}\;\exp(\pm i\psi )[\begin{array}{c} 0\\ 0\\ 1 \end{array}] \end{array}$$

**FIGURE 1:**
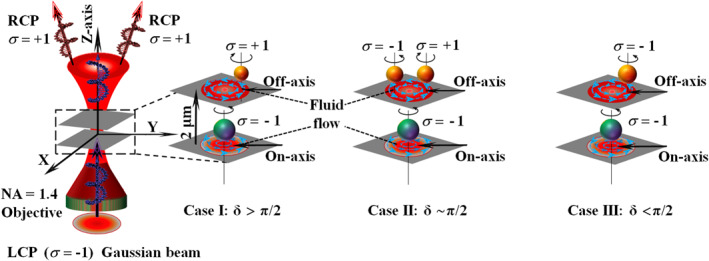
Schematic illustration of direct and indirect helicity (σ) generation. The direct helicity follows the input helicity and drives fluid flow that causes secondary particles to orbit around it. Indirect helicities appear at off‐axis positions and depend on the linear retardance (δ) of the central particle. Case I (δ>π/2): secondary particles spin opposite to the central particle. Case II (δ∼π/2): secondary particles may spin either in the same or opposite direction, depending on off‐axis spatial positions. Case III (δ<π/2): secondary particles spin in the same direction as the central particle.

From Equation (2), the coefficient of the first term, $I_{00}$, corresponds to the primary helicity of the input RCP/LCP Gaussian beam (same helicity). Transfer of this component to the birefringent LC particle induces spinning in the direction of the circulating electric field (see Figure 1), as observed for the primary particle trapped at the beam center. The coefficient of the second term, $I_{02}$, carries OAM of ±2ℏ and represents helicity flipping (σ=±1→∓1, i.e., opposite helicity). The coefficient of the third term, $I_{01}$, carries OAM of ±ℏ and describes conversion from circular to linear polarization (σ=±1→0), representing the longitudinal field component. Notably, the first term $\left(I_{00}\right)$ dominates, accounting for more than 85% of the field energy, and drives the motion of the centrally trapped primary LC particle. The last two terms, however, govern the strength of SOI in the nonparaxial regime (plots of their squared moduli are provided in Figure S1). Spin–orbit conversion here originates from the adiabatic evolution of the k‐vector and polarization during tight focusing, giving rise to the momentum‐dependent PB phase [26, 29]. The gradient of this phase leads to the emergence of OAM (vorticity).

In addition, the focused spin‐polarized beam interacting with the birefringent central LC particle produces scattered light containing indirectly generated helicities. These scattered components include both same and opposite helicities, depending on the birefringence of the central particle, and are governed by the Pancharatnam–Berry phase [26, 33, 34]. The out‐coupled light then dictates the helicity‐dependent spinning of orbiting secondary particles, reinforcing their synchronized revolution and rotation. Thus, the final expression for the output electric field emerging from the centrally trapped LC particle can be written as follows:

(3) $$\begin{array}{c} \left(\begin{array}{c} 1\\ \sigma i \end{array})\overset{Anisotropic}{\underset{Medium\left(LC\right)}{\to }}\cos\;\delta /2\cdot (\begin{array}{c} 1\\ \sigma i \end{array})-ie^{2\sigma i\psi }\cdot \sin\;\delta /2(\begin{array}{c} 1\\ -\sigma i \end{array}\right) \end{array}$$

where σ=±1 denotes the helicity of the focused field, ψ is the director orientation of the centrally trapped LC particle, and δ represents its linear retardance. In Equation (3), the first term on the right‐hand side corresponds to the same‐helicity component, with a magnitude determined by cos(δ/2). The second term, on the other hand, arises as a consequence of extrinsic SOI, representing the opposite‐helicity component associated with the Pancharatnam–Berry phase $\left(e^{2\sigma i\psi }\right)$, with its magnitude determined by sin(δ/2). The relative strength of these two contributions governs how off‐axis secondary LC particles orbit around the centrally trapped one. Depending on the value of $\delta_{LC}$, the spinning behavior of the orbiting particles can be categorized as follows: Case I—When the linear retardance $\left(\delta_{LC}\right)$ of the centrally trapped primary LC particle exceeds π/2 (e.g., $\delta_{LC}=2>\pi /2$), the coefficient of the second term (sin(δ/2)), which represents the opposite helicity, dominates. Consequently, secondary LC particles follow the opposite (or orthogonal) helicity with respect to the primary particle. Case II—When the linear retardance of the centrally trapped LC particle equals π/2 $\left(\delta_{LC}=\pi /2\right)$, the coefficients of the first term (cos(δ/2)) and the second term (sin(δ/2)) contribute equally. As a result, the off‐axis trapped LC particles may spin either clockwise or counterclockwise, depending on their spatial position. Case III—When the linear retardance of the centrally trapped LC particle is less than π/2 (e.g., $\delta_{LC}=1.2<\pi /2$), the coefficient of the first term (cos(δ/2)), which represents the same helicity, dominates. Therefore, the orbiting secondary LC particles follow the input (or same) helicity of the field. These effects are illustrated in Figure 1 and form the basis for understanding the collective spinning–orbiting dynamics observed in our experiments.

## 3 Results and Discussion

### 3.1 Resultant Mueller Matrix

To explain the generation of indirect helicities observed experimentally, we developed a Mueller‐matrix (MM) model. The tight focusing of spin‐polarized Gaussian beam from a cylindrical to a spherical symmetric system through an aplanatic lens (i.e., the paraxial to nonparaxial transformation) generates the intrinsic nature of SOI of light as a consequences of the gradient of momentum‐dependent PB phase as illustrated by Equation (2). However, when tightly focused spin‐polarized light interacts with the anisotropic medium of an LC particle, a Pancharatnam–Berry phase arises as a consequence of extrinsic SOI, as described by Equation (3) [14, 26, 34]. The combined effects of tight focusing and beam propagation through the anisotropic LC medium act as successive polarization‐transforming events, represented by the resultant 4×4 Mueller matrix $M_{\mathrm{res}}\left(d,\delta ,\psi \right)=M_{TF}\left(d_{TF},\delta_{TF},\psi \right)\cdot M_{LC}\left(d_{LC},\delta_{LC},\psi \right)$, where $M_{\text{TF}}$ MM of tight focusing and $M_{\text{LC}}$ MM of the bipolar LC particle. Following this, we numerically computed the 4 × 4 MM $\left(M_{TF}\left(d_{TF},\delta_{TF},\psi \right)\right)$ for the tight focusing of the Gaussian beam using a 2 × 2 Jones matrix of the transverse electric field, which revealed a characteristic form of a linear diattenuator $d_{TF}$ (partially polarizing), and a retarder $\delta_{TF}$ (wave plate) with azimuthal orientation ψ (see Supporting Information S1: Section 2.1 for more details). We then experimentally recorded the MM of bipolar LC particles (see Figure S9, Section 4: Experimental Method) and, using LU–Chipman decomposition [34, 35], we extracted the polarization parameters: linear diattenuation $d_{LC}$ and linear retardance $\delta_{LC}$ (detailed procedures are mentioned in Section 6.3). Measurements across multiple particles revealed retardance values spanning $\delta_{LC}∼$ 0.5–3, which we classified into three regimes: $\delta_{LC}<\pi /2$, $\delta_{LC}=\pi /2$, and $\delta_{LC}>\pi /2$. Using these values, we constructed the customized MM of LC $M_{\text{LC}}\left(d_{LC},\delta_{LC},\psi \right)$ and, by multiplying with $M_{\text{TF}}$, obtained the resultant $M_{\text{res}}\left(d,\delta ,\psi \right)$. The detailed calculations and the 4× 4 Mueller matrices of $M_{TF}$, $M_{LC}$, and $M_{\mathrm{res}}$ are provided in Supporting Information S1: Sections 2.1–2.3. We then proceed to calculate the resultant Stokes vector using the relation $S^{\mathrm{out}}=M_{res}S^{in}$, which captures the polarization distribution of the scattered (emerging) light and reveals the generation of indirect helicities. Where $M_{res}$ is the 4 × 4 MM representing the composite effect, and $S^{in}$ and $S^{\mathrm{out}}$ are the input and output Stokes vectors, respectively.

### 3.2 Resultant Stokes Vector

The study of SAM and its relationship with the polarization of light reveals fascinating dynamics in tightly focused beams on LC particles. The SAM (S), which is intrinsic in nature, depends entirely on the polarization of the beam and can be described by the following equation: $S\propto \text{Im}\;\left[\epsilon \left(E^{*}\times E\right)+\mu \left(H^{*}\times H\right)\right]$, with ϵ as the electric permittivity and μ as the magnetic permeability [3, 7, 36]. The S3 component of the Stokes vector, which illustrates the distribution of right and left circular polarization (or the difference in $\sigma_{+}$ and $\sigma_{-}$ helicity of light), is proportional to $i\left(\langle E_{y}E_{x}^{*}\rangle -\langle E_{x}E_{y}^{*}\rangle \right)$, where $\langle \text{…}\rangle$ represents the temporal average of the electric field [26]. Therefore, the longitudinal SAM (LSAM) density and the S3 component of the Stokes vector are equivalent. Following this analogy, we calculated the resultant Stokes vector elements S0, S1, S2, and S3 for input LCP and RCP light $\left(S_{\mathrm{LCP∕RCP}}^{in}={\left[\begin{array}{cccc} 1 & 0 & 0 & \mp 1 \end{array}\right]}^{T}\right)$ using the resultant MM of the composite effect $M_{\text{res}}$ at a fixed linear diattenuation value of 0.02 and different linear retardance $\left(\delta_{LC}\right)$ values. To analyze the effect of LSAM of scattered field (i.e., same and opposite indirectly generated helicity of light), we plotted the resultant S3 component of the Stokes vector for input LCP and RCP light at linear retardance values $\delta_{LC}<\pi /2$, $\delta_{LC}=\pi /2$, and $\delta_{LC}>\pi /2$. Here, we present only the resultant S3 component of the Stokes vector, whereas the other resultant Stokes vector elements (S0, S1, S2) corresponding to $\delta_{LC}<\pi /2$, $\delta_{LC}=\pi /2$, and $\delta_{LC}>\pi /2$ are detailed in Supporting Information S1: Section 2.4.

**FIGURE 2:**
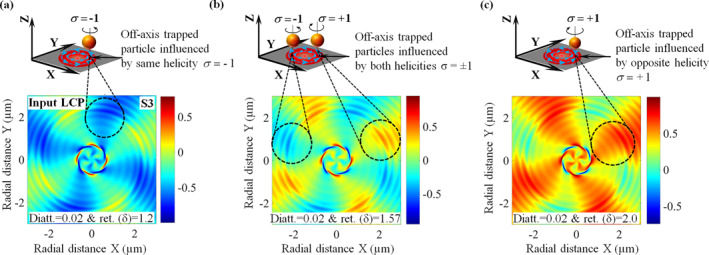
The distribution of the resultant S3 component of the Stokes vector for tightly focused circularly polarized light incident on an LC particle at a fixed linear diattenuation value of 0.02 and different linear retardance values. This illustrates that indirect helicities appear at off‐axis positions and depend on the linear retardance (δ) of the central particle, which influences the secondary particles. The extrema values of S3 are different and explicitly depend on the linear retardance $\delta_{LC}$ of the central LC particle. Panels (a–c) show the results for left‐circularly polarized light with δ=1.2, 1.57, and 2.0, respectively. For δ=1.2<π/2, the indirect same helicity is dominant. For δ=1.57≈π/2, spatially resolved indirect same and opposite helicities coexist with equal strength. However, for δ=2.0>π/2, the indirect opposite helicity dominates at off‐axis positions.

In Figure 2a–c, we show the distribution of the resultant S3 component of the Stokes vector for tightly focused LCP light on primary LC particle at a fixed linear diattenuation $d_{LC}=0.02$ and linear retardance values of 1.2, 1.57, and 2.0, respectively, whereas the corresponding opposite distributions for RCP input are provided in Figure S6. In our convention, positive $\left(\sigma_{+}\right)$ and negative $\left(\sigma_{-}\right)$ values of S3 represent clockwise and counter‐clockwise spinning of the LC particle about the z‐axis. The results corroborate our theoretical predictions: when $\delta_{LC}<\pi /2$ $\left(\delta_{LC}=1.2\right)$, the indirect same‐helicity component dominates at the off‐axis positions (see Figure 2a), and therefore the central and off‐axis particles spin in the same direction, as expected from Equation (3); when $\delta_{LC}=\pi /2$, the coefficients of both “same” and “opposite” indirect helicities ($\sigma_{-}$/$\sigma_{+}$) components are equal as describe by Equation (3), yielding spatially separated regions of $\sigma_{-}$ and $\sigma_{+}$ (see Figure 2b), so that the off‐axis particle spin direction depends on its spatial location (counter‐clockwise in $\sigma_{-}$ and clockwise in $\sigma_{+}$); and when $\delta_{LC}>\pi /2$ ($\delta_{LC}=2$), the indirect opposite‐helicity component dominates at the off‐axis position as illustrated in Figure 2c, so that the orbiting secondary particles spin in the opposite direction to the centrally trapped primary one. These numerical simulations validate the generation of indirect helicities arising from extrinsic SOI effects described by Equation (3) and provide the basis for the experimental observations presented in the following.

### 3.3 Experimental Results

We now study instances where the secondary particles orbiting around the primary particle also spin. Such simultaneous spinning–and–orbiting of particles—reminiscent of planetary motion—is rarely observed at the microscale. In our system, the combined effects of tight focusing and the anisotropic medium of the LC particle generate both same‐ and opposite‐helicity components (indirectly generated helicities) at off‐axis positions, depending on the linear retardance of the primary particle, as predicted by our simulations in Figure 2a–c. The field emerging from the primary LC particle couples to outer (off‐axis) secondary LC particles at different z‐planes due to the diverging nature of the scattered field, inducing them to spin either in the same or in the opposite direction through extrinsic SOI. As a result, we observe planetary‐like dynamics, with primary and secondary particles simultaneously spinning and orbiting under input LCP or RCP excitation. However, when the LC particles are spatially separated from one another, the transfer of indirect helicities is not feasible; in this case, only the direct helicity acts on both particles. This scenario is discussed in detail in Supporting Information S1: Section 3.1 (see Video S5).

**FIGURE 3:**
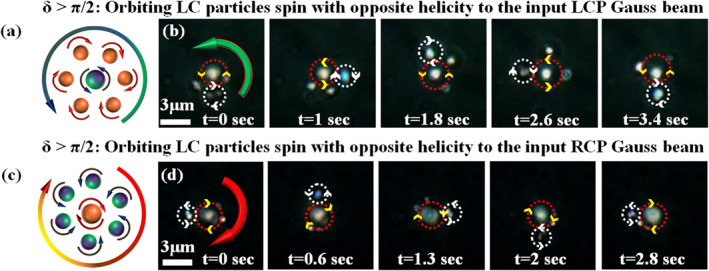
(a, c) Cartoon representations of the primary LC particle trapped on‐axis and the secondary LC particle trapped off‐axis, showing spinning and orbiting dynamics. (b, d) Time‐lapse frames from Videos S1 and S2 for input LCP and RCP light, respectively. The on‐axis primary particle (within red dotted circle) spins counterclockwise (b) and clockwise (d) due to direct helicity transfer, while the off‐axis secondary particle (within white dotted circle) orbits and spins in the opposite direction due to indirect helicity transfer.

Cartoon representations of the primary particle spinning on‐axis and the secondary particles undergoing combined spinning and orbiting motion at off‐axis positions (planetary‐like motion) are shown in Figure 3a,c for input LCP and RCP beams, respectively. The outer deep green/red arrows denote fluid (or spin) flow generated by the centrally spinning primary particle, whereas the circular arrows associated with the secondary particles indicate their spinning directions. Experimental observations are presented in Figure 3b,d (time‐lapse images from Videos S1 and S2). Details of the experimental setup, sample preparation, and measurement procedure are provided in Section 6. The on‐axis primary particle (within the red dotted circle) spins counterclockwise/clockwise at high speed for an input LCP/RCP beams, confirmed by the motion of surrounding orbiting particles or by reducing the input beam power. However, the off‐axis secondary particle (within the white dotted circle) orbits due to fluid flow and spins in the opposite sense (clockwise/counterclockwise) relative to the central spinning particle at a different z‐plane. This occurs because the linear retardance of the primary particle is greater than π/2 $\left(\delta_{LC}=2>\pi /2\right)$. Consequently, the coefficient of the second (opposite‐helicity) term in Equation (3), sin(δ/2), dominates, causing the orbiting secondary particle to spin opposite to the central primary particle, as illustrated in Figure 2c. However, the time series of rotational dynamics shown in Figure 3b,d may appear different between left‐ and right‐circularly polarized input beams. This arises from unavoidable experimental variations in particle size, trapping z‐plane, and the resulting coupling between primary and secondary particle motions. Moreover, secondary particles can exhibit complex dynamics involving simultaneous rotation and revolution, or counter‐rotations, which makes precise control of all parameters challenging. In addition, the rotation rate also depends on the linear retardance $\left(\delta_{\text{LC}}\right)$ of the central particle, which may be different for different experimental realizations. Note that in this regime, the primary particle effectively acts as a q‐plate due to the azimuthally varying orientation of its anisotropic LC molecular directors [33, 37, 38]. When an RCP (LCP) Gaussian beam passes through such a q‐plate, it is converted into an LCP (RCP) optical vortex beam through spin–orbit conversion [38].

**FIGURE 4:**
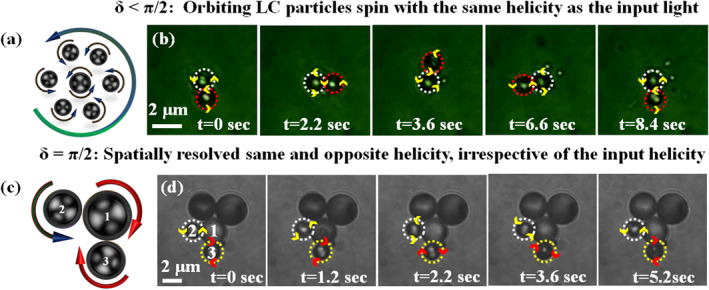
(a) Cartoon illustrating the on‐axis primary LC particle spinning and the off‐axis secondary LC particle both spinning and orbiting in the counterclockwise direction. (b) Time‐lapse frames from Video S3 for input LCP light, showing the on‐axis primary particle (within the white dotted circle) spinning counterclockwise due to direct helicity transfer, whereas the off‐axis secondary particle (within the red dotted circle) orbits counterclockwise due to the induced fluid flow and simultaneously spins in the same direction at a different z‐plane, driven by the transfer of indirect helicity. (c) Cartoon illustrating spatially resolved opposite spinning of secondary LC particles at off‐axis positions. (d) Time‐lapse frames from Video S4 for input RCP light, showing that the LC particles within the white and yellow dotted circles spin counterclockwise and clockwise, respectively, due to spatially resolved indirect helicity transfer.

In another case, when the linear retardance $\left(\delta_{LC}\right)$ of the primary particle is less than π/2 $\left(\delta_{LC}=1.2<\pi /2\right)$, the coefficient of the first term in Equation (3), cos(δ/2) (same helicity), dominates, causing the orbiting secondary particle to spin in the same direction as the primary, as predicted by our theory and numerical simulations (see Figure 2a). This behavior is illustrated in Figure 4a, where a cartoon shows the primary LC particle spinning on‐axis, whereas the secondary particle orbits off‐axis, both following the same helicity dynamics. Experimentally, as shown in Figure 4b (time‐lapse images from Video S3), the primary particle (within white dotted circle) spins counterclockwise at high speed, confirmed either by the motion of the surrounding orbiting particles or by reducing the input beam power of an LCP Gaussian beam. The secondary particle (within the red dotted circle) orbits counterclockwise due to the induced fluid flow and simultaneously spins in the same direction at a different z‐plane, driven by the transfer of indirect helicity.

However, when the linear retardance $\left(\delta_{LC}\right)$ of the primary particle is close to π/2 $\left(\delta_{LC}∼\pi /2\right)$, the coefficients of the first and second terms in Equation (3) (cos(δ/2) and sin(δ/2), corresponding to same and opposite indirect helicity) contribute equally. In this case, we observe spatially resolved same‐ and opposite‐helicity motions of the secondary particles, depending on their off‐axis trapping positions around the primary LC particle, as shown schematically in Figure 4c, where particle No. 2 spins counterclockwise (opposite helicity) and particle No. 3 spins clockwise (same helicity) with respect to the centrally trapped particle No. 1. Experimentally, as shown in Figure 4d (time‐lapse images from Video S4), the secondary particles in the white and yellow dotted circles spin counterclockwise (opposite helicity) and clockwise (same helicity), respectively. This simultaneous counterclockwise and clockwise spinning of secondary LC particles arises from the isotropic inhomogeneous distribution of σ=−1 and σ=+1 indirect helicities at off‐axis, as shown in Figure 2b. The centrally trapped large primary particle (particle No. 1) also spins slowly in a clockwise direction, which we verified directly through the microscope eyepiece (consistent with the input helicity of light). Two additional large secondary particles are also trapped off‐axis due to the intensity gradient. One of them spins very slowly, although its direction of rotation could not be determined from Video S4. In all these cases, we utilized the bipolar nature of the LC particles, with asymmetrically oriented anisotropic directors; thus, the bipolar configuration of LC directors is spin‐responsive. In contrast, the radial configuration of LC directors is spin‐irresponsive due to their symmetrically oriented anisotropic directors. These studies are described in detail in Supporting Information S1: Section 3 (see Videos S6 and S7). It is noteworthy that the spinning directions of the particles were confirmed using our cross‐polarization detection mechanism (details are provided in Section 6 and in ref. [3]).

## 4 Conclusion

In conclusion, we have demonstrated a mechanism by which extrinsic SOI can be harnessed to generate indirect helicity. This process induces rotational dynamics in microscopic particles, resulting in planetary‐like motion at the microscale. In our experiments, a primary birefringent LC particle spins at the center of a spherically aberrated optical trap, whereas secondary LC particles simultaneously orbit and spin around it. Thus, we show that the primary particle efficiently converts the longitudinal spin angular momentum of the input light into an indirectly generated helicity, enabling the controlled rotational motion of the secondary particles without the need for mechanical components such as gear structures. Our numerical simulations and experiments reveal that the combined effects of tight focusing and scattering from anisotropic LC media fundamentally generate indirect helicities in the nonparaxial regime, manifesting as same‐ or opposite‐helicity components in particle dynamics. The primary particle, governed by the input circular polarization, induces fluid flow that drives the orbital motion of nearby secondary particles, whose spinning direction depends entirely on the linear retardance $\delta_{LC}$ of the primary LC particle. Specifically, when $\delta_{LC}>\pi /2$, the secondary particles spin in the opposite direction to that of the primary; whereas when $\delta_{LC}<\pi /2$, both spin in the same direction; and finally, when $\delta_{LC}∼\pi /2$, spatially resolved regions of same and opposite helicity coexist. Our findings establish a clear framework for helicity‐dependent collective dynamics in optical tweezers and demonstrate a gear‐free optical platform for spin‐engineered micromotors capable of coordinated multi‐body motion, thereby opening pathways towards harvesting SOI for developing next‐generation light‐driven micromechanical systems.

## 5 Numerical Methods

**FIGURE 5:**
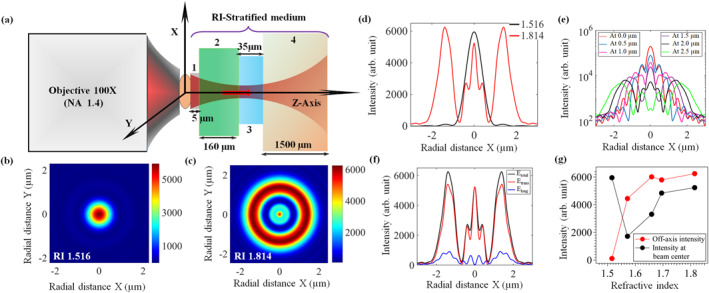
(a) Schematic of the refractive‐index–stratified medium used in both numerical simulations and experiments. Numerical analysis of the intensity distribution at z=2μm from the focus of a high‐NA objective lens for an input circularly polarized Gaussian beam. (b and c) Total intensity distribution of an RCP/LCP Gaussian beam at z=2μm from the focus for RI = 1.516 (matched coverslip) and RI = 1.814 (mismatched coverslip), respectively. (d) Line profiles of (b and c), showing the deviation from a Gaussian profile when changing from a matched to a mismatched RI coverslip. (e) Line profiles (log scale) of the intensity distribution at different z‐planes for RI = 1.814, illustrating the progressive loss of Gaussian nature away from the focus. (f) Comparison of transverse, longitudinal, and total electric field intensity distributions in the xy‐plane for RI = 1.814 at z=2μm. (g) Variation of beam‐center intensity (black circles) and off‐axis intensity (red circles) as a function of refractive index at z=2μm.

In our experimental system, the output beam from the high NA objective lenses in the optical tweezers setup is passed through a RI‐stratified medium as shown in Figure 5a. The laser beam of wavelength 671 nm is incident on the 100X oil immersion objective of NA 1.4 followed by (a) an oil layer of thickness around 5 μm and RI 1.516, (b) a 160 μm thick coverslip having RI varying between 1.516 and 1.814 (note that the case where the RI=1.516 is henceforth referred to as the “matched condition,” which is typically employed in optical tweezers to minimize spherical aberration effects in the focused beam spot, whereas the other values are referred to as a “mismatched” condition), (c) a sample chamber of an aqueous solution of LC particles/droplets in a water medium having a RI of 1.33 with a depth of 35 μm, and finally (d) a glass slide of RI 1.516 whose thickness we consider to be semi‐infinite (1500 μm) (see Figure 5a). In the simulation, the origin of coordinates is taken inside the sample chamber at an axial distance of 5 μm from the interface between the sample and the coverslip. Thus, the objective‐oil interface is at −170 μm, the oil‐cover slip interface is at −165 μm, the cover slip‐sample chamber interface is at −5 μm, and the sample chamber‐glass slide interface is at +30 μm. At the paraxial to nonparaxial transformation boundary, we decompose the incident polarized rays $E_{\mathrm{inc}}$ into s‐polarization and p‐polarization. For each polarization state, we account for the corresponding Fresnel transmission coefficients $T_{s}$ and $T_{p}$, as well as the reflection coefficients $R_{s}$ and $R_{p}$ at each interface of the RI‐stratified medium [3, 29, 31, 32].

### 5.1 Numerical Simulations of Intensity Distribution

Figure 5a shows a schematic representation of the numerical and experimental system. Following this approach, we performed numerical simulations to analyze the radial intensity distribution at 2μm above the focal plane for both matched (RI = 1.516) and mismatched (RI ≠ 1.516) refractive indices (RI) of the stratified medium. Figure 5b–g present the intensity distribution of a circularly polarized (RCP/LCP) Gaussian beam. For the matched condition (RI = 1.516), the intensity distribution retains a purely Gaussian profile as shown in Figure 5b. However, for the mismatched condition (RI = 1.814), spherical aberration distorts the beam, resulting in a non‐Gaussian distribution Figure 5c. In Figure 5d, the line profiles corresponding to Figure 5b,c highlight the deviation from a Gaussian profile under mismatched RI. The broadened and distorted profile enables the trapping of multiple LC particles simultaneously in experiments. Figure 5e shows line profiles of the intensity distribution at different axial planes (z) for RI = 1.814. As we move away from the focus, off‐axis intensity lobes emerge, providing sufficient spatial extent in the transverse plane to trap multiple particles.

In Figure 5f, we separately plot the transverse and longitudinal components of the electric field intensity distribution and compare them with the total electric field intensity. The longitudinal component $\left(E_{\text{long}}\right)$ is primarily concentrated at the off‐axis position due to the first‐order Bessel function $\left(J_{1}\right)$ embedded in the $I_{01}$ coefficient, contributing about 10%–15% of the total intensity. By contrast, the transverse intensity profile $\left(E_{\text{trans}}\right)$, arising from $I_{00}$ and $I_{02}$, is distributed at both the beam center and the off‐axis region, producing a central maximum as well as an annular intensity ring (because the zero‐order Bessel function $J_{0}$ embedded in $I_{00}$ has a nonvanishing value on‐axis). The transverse components account for about 85%–90% of the total intensity $\left(E_{\text{total}}\right)$, as illustrated in Figure 5f. Therefore, when an LC particle is trapped at the beam center, it follows the input helicity of the light, as the $I_{00}$ coefficient in Equation (2) corresponds to the input RCP/LCP helicity. In order to obtain the most optimized experimental conditions, we plotted the variation of intensity at the beam center (on‐axis) and off‐axis positions as a function of the refractive index (RI) in Figure 5f. It is noteworthy that under the matched condition of RI 1.516, at 2 μm away from the focus, most of the intensity is concentrated at the beam center, with less intensity distributed off‐axis. This indicates that the Gaussian beam retains its Gaussian nature as shown in Figure 5b. In contrast, under the mismatched condition (RI = 1.814), the on‐axis and off‐axis intensities become comparable, and the resulting profile resembles that of a tightly focused first‐order radially polarized vector beam, as reported in refs. [3, 39, 40, 41]. This configuration enables stable trapping of particles in both the central and annular regions.

## 6 Experimental Methods

### 6.1 Experimental Setup

**FIGURE 6:**
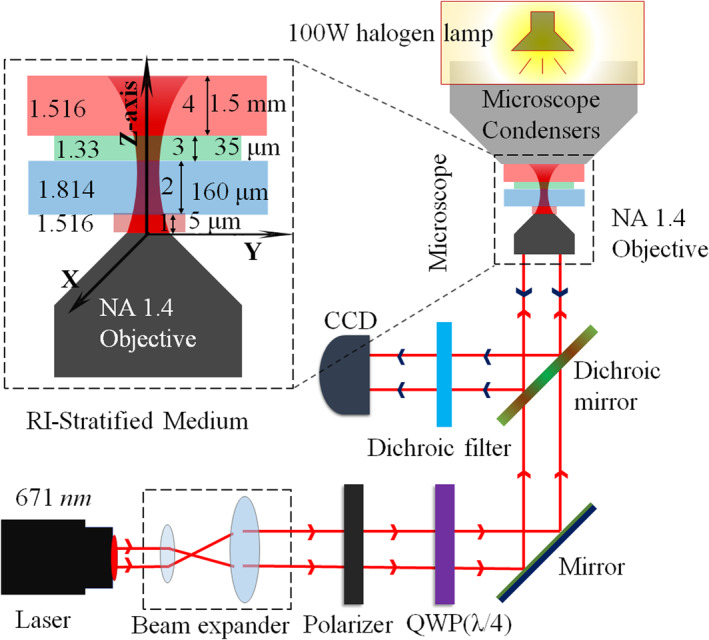
Schematic diagram of our experimental setup. The ray diagram illustrates the generation and tight focusing of circularly polarized Gaussian beams in optical tweezers, while the inset depicts the RI‐stratified medium used in both the numerical simulations and the experiment.

The schematic of our optical tweezers setup is shown in Figure 6. We employ a standard inverted microscope (Carl Zeiss Axiovert.A1) with a 100× oil‐immersion objective (NA 1.4) and a solid‐state laser (Lasever, 671 nm, 350 mW) coupled to the back port [3, 42]. A wire‐grid polarizer generates linearly x‐polarized light, which is converted to right‐ or left‐circular polarization (RCP/LCP) by a quarter‐wave plate (QWP, 671 nm) oriented at 45° or 135°. Nematic LC colloids are used as probe particles; their optical anisotropy and birefringence enable spin angular momentum transfer from the beam [3, 6, 27]. The circularly polarized Gaussian beam is tightly focused into a refractive‐index–stratified medium (see Figure 6). Mismatched coverslips (RI 1.814) enhance spherical aberrations, broadening the focal field distribution (see Figure 5c) [3, 29], which facilitates trapping large or multiple LC particles and probing longitudinal SAM (LSAM). The sample chamber, formed by a coverslip and glass slide, contained 20–30 μL of LC dispersion (mean size 2–8 μ *m*, ∼20% standard deviation).

We collected both forward‐transmitted light (from the lamp) and back‐reflected light from LC particles to track their spin and orbital motion. LSAM transfer to particles trapped on‐axis or off‐axis (in the annular intensity ring, see Figure 5c) was optimized by adjusting the z‐focus. To characterize yaw‐type rotation induced by LSAM, we employed a cross‐polarization scheme [3, 43], placing orthogonal polarizers at the input and output. This produced polarization‐dependent intensity lobes across LC surfaces, revealing their scattering and rotational behavior.

### 6.2 Sample Preparation

We used liquid‐crystal colloids as probe particles, which are optically anisotropic and birefringent, enabling transfer of SAM from the beam to the particles [3, 6, 27, 37]. The colloids were derived from nematic liquid crystal 5CB (4′‐Pentyl‐4‐biphenylcarbonitrile) [42]. A dispersed solution was prepared by mixing 5μl of 5CB with 100 mL of deionized water, followed by centrifugation for 2–5 min. This produced a concentrated dispersion of LC colloids (droplets) with radii of 1–8μ *m*. The concentration could be adjusted by diluting with deionized water. The solution exhibited diverse LC director configurations, including radial, pre‐radial, twisted radial, monopolar, bipolar, twisted bipolar, and sunset structures [37, 44, 45]. By adding 100–150μl of polyvinyl alcohol (PVA), we obtained predominantly bipolar configurations, which were verified using cross‐polarization imaging [33, 37].

The dielectric constants $\epsilon_{\|}$ and $\epsilon_{⊥}$ are related to the ordinary and extraordinary refractive indices $n_{\|}$ and $n_{⊥}$ of the birefringent particle through the relationships $n_{\|}^{2}=\epsilon_{\|}$ and $n_{⊥}^{2}=\epsilon_{⊥}$ [27]. The optical birefringence Δn value ranges from 0.1 to 0.4 for the nematic LC particles, resulting in a linear retardance $\left(\delta_{LC}\right)$ value ranging from 0.5 to 3 [27, 45].

### 6.3 Mueller Matrix Measurement Procedure

**FIGURE 7:**
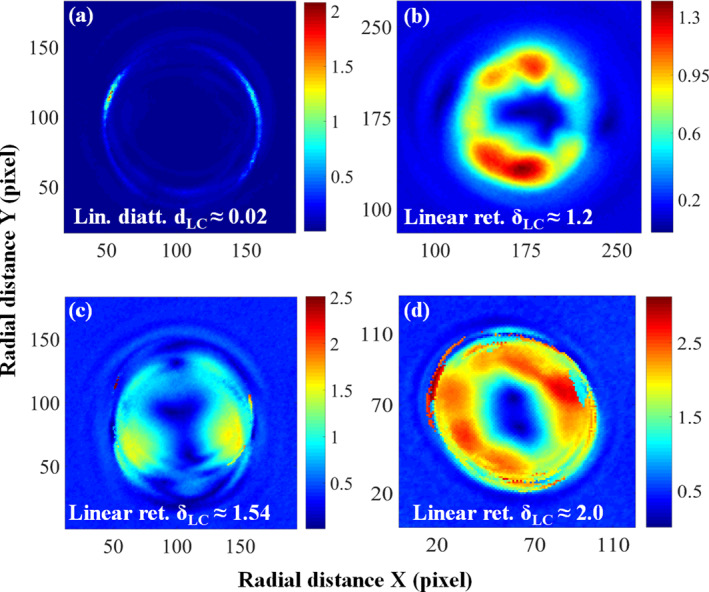
The polarization parameters of LC particles (a) linear diattenuation $d_{LC}\approx 0.02$. The linear retardance (b) $\delta_{LC}\approx 1.2<\pi /2$ (c) $\delta_{LC}\approx 1.54⪅\pi ⁄2$ (d) $\delta_{LC}\approx 2.0>\pi /2$.

To determine the Mueller matrix (MM) of LC particles, we used a polarization state generator (PSG) at the input of the microscope and a polarization state analyzer (PSA) at the output. The PSG, consisting of a rotatable polarizer and a quarter‐wave plate (671 nm), generated six different linear and circular polarization states. Collimated white light from the microscope's inbuilt illumination source (100 W halogen lamp) passed through the PSG and illuminated the birefringent LC sample. The scattered light was collected by a microscope objective (Carl Zeiss Axiovert.A1, NA 1.4) and analyzed by the PSA, which comprised a quarter‐wave plate and a linear polarizer. For each PSG state, six PSA measurements were taken, giving 36 polarization‐resolved projective measurements. These were used to construct the full 4×4 Mueller matrix (see Table S1) [26, 34]. Using LU–Chipman decomposition [34, 35, 46], we extracted polarization parameters including linear diattenuation $\left(d_{LC}\right)$ and linear retardance $\left(\delta_{LC}\right)$ as illustrated in Figure 7a–d. We collected multiple sets of experimental data and observed that our sample exhibited a relatively constant diattenuation value of approximately d≈0.02, as shown in Figure 7a. However, the linear retardance $\left(\delta_{LC}\right)$ varied between 0.5 and 3, which we categorized into three distinct groups: $\delta_{LC}<\pi /2$, $\delta_{LC}\approx \pi /2$, and $\delta_{LC}>\pi /2$. Figure 7b–d display the nonuniform distribution of linear retardance values for these three groups, respectively. Consequently, the LC particles are nonabsorbing (very low diattenuation) and primarily act as retarders, generating a phase difference between two orthogonal linear polarization states.

## Author Contributions

R.N.K. set up and performed the experiments and carried out the numerical simulations. J.K.N. and N.G. assisted in the numerical simulations of the Mueller matrix and Stokes vectors. R.N.K., S.D.G., N.G., and A.B. conceived the idea, conceptualized the study, and analyzed the experimental findings. R.N.K. and A.B. mainly wrote the manuscript, while S.D.G. revised and corrected the final draft. All authors have accepted responsibility for the entire content of this manuscript and approved its submission. .

## Funding

R.N.K. acknowledges support from the IPh.D. research fellowship at IISER Kolkata. This work was also supported by SERB, DST, Government of India (Project No. EMR/2017/001456).

## Conflicts of Interest

The authors declare no conflicts of interest.

## Supporting information


Supporting Information S1

Supporting Information S2

## Data Availability

The datasets produced and/or examined in this study can be obtained from the corresponding authors upon reasonable request.
