# Supplementary material for: Harvesting the Spin–Orbit Interaction of Light to Generate Helicity‐Dependent Complex Rotational Motion in Optically Trapped Mesoscopic Matter
Source: Nanophotonics. 2026 Feb 24;15(5):e70034. doi: 10.1002/nap2.70034 (PMC12965037; doi:10.1002/nap2.70034)
Supplement: Supplementary file 1 — Supporting Information S1 [file NAP2-15-e70034-s001.zip › Supplementary_Material_updated.pdf]

Supplementary Material

Ram Nandan Kumar*, Jeeban Kumar Nayak, Subhasish Dutta Gupta, Nirmalya Ghosh, and Ayan Banerjee

Harvesting the spin–orbit interaction of light to generate helicity-dependent complex rotational motion in optically trapped mesoscopic matter

1 THEORETICAL CALCULATIONS

We use the Debye-Wolf theory or the angular spectrum method to analyze the spin-orbit interaction (SOI) of light in the non-paraxial regime. In this approach, the incoming collimated Gaussian beam is decomposed into a superposition of plane waves, each associated with a distinct spatial harmonic component (k-vector). The angular spectrum integral for a tightly focused fundamental Gaussian beam passing through a high numerical aperture (NA) objective lens can be expressed as [1–3]:

$$\vec{E}(\rho,\psi,z) = i\frac{kfe^{-ikf}}{2\pi}\int_0^{\theta_{\max}}\int_0^{2\pi}\vec{E}_{\rm res}(\theta,\phi)\,e^{ikz\cos\theta}\,e^{ik\rho\sin\theta\cos(\phi-\psi)}\,\sin\theta\,d\phi\,d\theta. \tag{1}$$

Here, $\vec{E}_{\rm res}(\theta,\phi)$ represents the resultant electric field in the refractive index (RI)-stratified medium, comprising a mixture of both forward- and backward-propagating waves, f is the focal length of the lens, k is the wave vector, and $\theta_{\max} = \sin^{-1}(\mathrm{NA}/n)$ is the maximum angle determined by the numerical aperture of the objective lens. n denotes the RI of the medium, while θ and ϕ refer to the polar angle relative to the z-axis and the azimuthal angle relative to the x-axis, respectively, in the cylindrical (or spherical) coordinate system.

The angular spectrum method operates in the frequency domain, where it calculates the Fourier transform (FT) of the input field $\vec{E}_{\rm inc}$ and then multiplies it by a transfer function. The desired output field $\vec{E}_{\rm res}$ is then obtained by taking the inverse FT. The transfer function accounts for the transformation from cylindrical to spherical coordinates. Before focusing, the incoming collimated Gaussian beam exhibits cylindrical symmetry. After tight focusing through an aplanatic lens or a high NA objective lens, the beam follows spherical symmetry. Therefore, at the transition from the paraxial to the non-paraxial regime, a transfer function representing the coordinate transformation is required.

The transfer function is given by $A = R_z(\phi)TR_y(\theta)R_z(-\phi)$, where R_z and R_y are SO(3) rotation matrices. Since the RI-stratification of the medium causes the field propagation to depend on the input

***Corresponding author: Ram Nandan Kumar,** Structured Light Laboratory, School of Physics, University of the Witwatersrand, Johannesburg 2000, South Africa, Department of Physical Sciences, Indian Institute of Science Education and Research Kolkata, Mohanpur-741246, West Bengal, India E-mail: ram.kumar@wits.ac.za;
Jeeban Kumar Nayak, Department of Physical Sciences, Indian Institute of Science Education and Research Kolkata, Mohanpur-741246, West Bengal, India
Subhasish Dutta Gupta, Tata Institute of Fundamental Research, Hyderabad, Telangana 500046, India
Nirmalya Ghosh, Department of Physical Sciences, Indian Institute of Science Education and Research Kolkata, Mohanpur-741246, West Bengal, India
Ayan Banerjee, Department of Physical Sciences, Indian Institute of Science Education and Research Kolkata, Mohanpur-741246, West Bengal, India

polarization, the Fresnel transmission coefficients T_s and T_p and the Fresnel reflection coefficients R_s and R_p are incorporated, considering both s- and p-polarizations ($E_{inc} = E^s_{inc} + E^p_{inc}$). For backward-propagating waves, the transfer function A is modified by replacing θ with $\pi - \theta$, and the Fresnel reflection coefficients R_s and R_p are used instead of the transmission coefficients. The resultant and incident electric fields are thus related through the transfer function A as

$$\mathbf{E}_{res}(\theta,\phi) = A\mathbf{E}_{inc}(\theta,\phi) \tag{2}$$

where, the T and R matrices are given by:

$$T = \begin{pmatrix} T_p & 0 & 0 \\ 0 & T_s & 0 \\ 0 & 0 & T_p \end{pmatrix}; R = \begin{pmatrix} -R_p & 0 & 0 \\ 0 & R_s & 0 \\ 0 & 0 & -R_p \end{pmatrix},$$

Note that we have $T_i^{(1,j)} = \dfrac{E^j_{i+}}{E^1_{i+}}; R_i^{(1,j)} = \dfrac{E^j_{i-}}{E^1_{i+}}$;. Here, i specifies the polarization (s and p), $+/-$ signifies a wave propagating forward and back-ward, respectively, and j in the superscript specifies the layer of the RI-stratified medium in which the optical tweezers (trapping laser) focus lies. we take into account the Fresnel transmission coefficients T_s and T_p, as well as the reflection coefficients R_s and R_p at the interface of the RI-stratified medium. For an input Gaussian TM_{00} mode [1, 2], we have

$$E_{\text{inc}} = E_0 e^{-f^2 \sin^2\theta / w_0^2} \tag{3}$$

Using equations 1, 2, and 3, we have calculated the 3X3 Jones matrix of tight focusing [1–3]. Now, the output and the input electric fields are related through a 3X3 Jones matrix of tight focusing as:

$$\begin{bmatrix} E^0_x \\ E^0_y \\ E^0_z \end{bmatrix}^{Gauss} = C \begin{bmatrix} I_{00} + I_{02}\cos 2\psi & I_{02}\sin 2\psi & 2iI_{01}\cos\psi \\ I_{02}\sin 2\psi & I_{00} - I_{02}\cos 2\psi & 2iI_{01}\sin\psi \\ -2iI_{01}\cos\psi & -2iI_{01}\sin\psi & I_{00} + I_{02} \end{bmatrix} \times \begin{bmatrix} E^i_x \\ E^i_y \\ E^i_z \end{bmatrix}, \tag{4}$$

where E^0 and E^i denote the output and input Jones polarization vectors, respectively, I_{00}, I_{01} and I_{02} are the Debye–Wolf (or diffraction) integrals for the transmitted and reflected waves, which are given as [1, 2].

$$\begin{aligned}
I^t_{00}(\rho) &= \int_0^{\theta_{\max}} E_{\text{inc}}(\theta)\sqrt{\cos\theta}\left(T_s^{(1,j)} + T_p^{(1,j)}\cos\theta_j\right) J_0\left(k_1\rho\sin\theta\right) e^{ik_j z\cos\theta_j}\sin\theta d\theta, \\
I^t_{01}(\rho) &= \int_0^{\theta_{\max}} E_{\text{inc}}(\theta)\sqrt{\cos\theta}\left(T_p^{(1,j)}\sin\theta_j\right) J_1\left(k_1\rho\sin\theta\right) e^{ik_j z\cos\theta_j}\sin\theta d\theta, \\
I^t_{02}(\rho) &= \int_0^{\theta_{\max}} E_{\text{inc}}(\theta)\sqrt{\cos\theta}\left(T_s^{(1,j)} - T_p^{(1,j)}\cos\theta_j\right) J_2\left(k_1\rho\sin\theta\right) e^{ik_j z\cos\theta_j}\sin\theta d\theta, \\
I^r_{00}(\rho) &= \int_0^{\theta_{\max}} E_{\text{inc}}(\theta)\sqrt{\cos\theta}\left(R_s^{(1,j)} - R_p^{(1,j)}\cos\theta_j\right) J_0\left(k_1\rho\sin\theta\right) e^{-ik_j z\cos\theta_j}\sin\theta d\theta, \\
I^r_{01}(\rho) &= \int_0^{\theta_{\max}} E_{\text{inc}}(\theta)\sqrt{\cos\theta}\left(R_p^{(1,j)}\sin\theta_j\right) J_1\left(k_1\rho\sin\theta\right) e^{-ik_j z\cos\theta_j}\sin\theta d\theta, \\
I^r_{02}(\rho) &= \int_0^{\theta_{\max}} E_{\text{inc}}(\theta)\sqrt{\cos\theta}\left(R_s^{(1,j)} + R_p^{(1,j)}\cos\theta_j\right) J_2\left(k_1\rho\sin\theta\right) e^{-ik_j z\cos\theta_j}\sin\theta d\theta,
\end{aligned} \tag{5}$$

Here, the superscripts t and r indicate the transmitted and reflected components, respectively. The functions J_0, J_1, and J_2 are the zero, first, and second-order Bessel functions of the first kind, respectively. The input Jones vectors for right circularly polarized (RCP) and left circularly polarized (LCP) light are given by $E^{\mathrm{i}}_{\mathrm{RCP/LCP}} = \begin{bmatrix} 1 & \pm i & 0 \end{bmatrix}^T$. Using Eq. 4, the output electric field for both RCP and LCP cases can be determined as

$$\begin{bmatrix} E_x^0 \\ E_y^0 \\ E_z^0 \end{bmatrix}^{Gauss}_{RCP/LCP} = \begin{bmatrix} I_{00} + I_{02}\cos 2\psi \pm iI_{02}\sin 2\psi \\ I_{02}\sin 2\psi \pm i\left(I_{00} - I_{02}\cos 2\psi\right) \\ -2iI_{01}\cos\psi \pm 2I_{01}\sin\psi \end{bmatrix} \tag{6}$$

Alternatively, We can decompose the output electric fields given in Eq. 6 for an input RCP or LCP Gaussian beam into their respective SAM and OAM components as

$$\begin{bmatrix} E_x^0 \\ E_y^0 \\ E_z^0 \end{bmatrix}^{Gauss}_{RCP/LCP} = I_{00}\begin{bmatrix} 1 \\ \pm i \\ 0 \end{bmatrix} + I_{02}\exp(\pm 2i\psi)\begin{bmatrix} 1 \\ \mp i \\ 0 \end{bmatrix} - 2iI_{01}\exp(\pm i\psi)\begin{bmatrix} 0 \\ 0 \\ 1 \end{bmatrix} \tag{7}$$

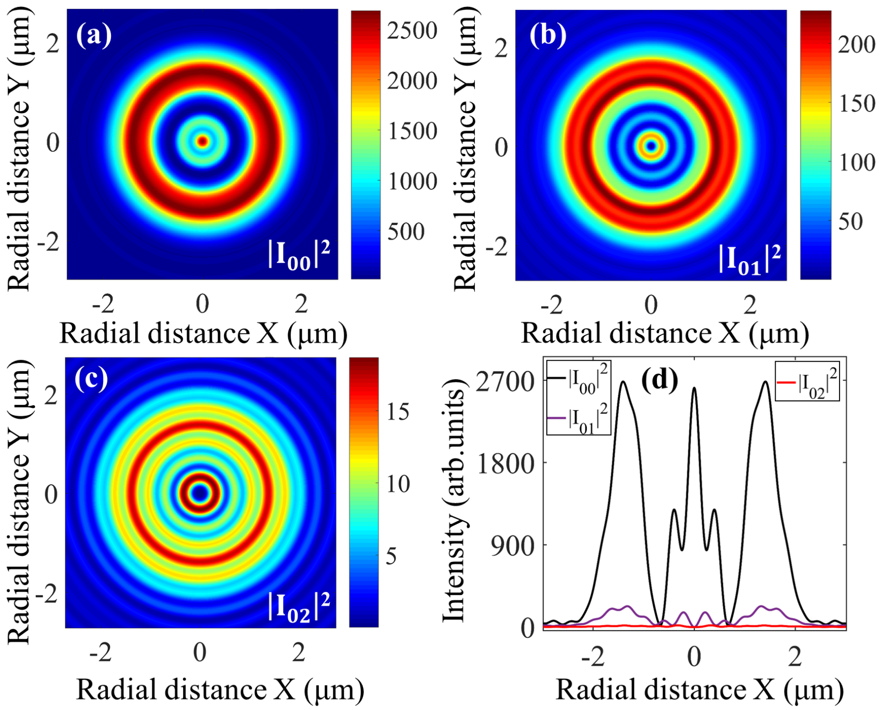

Fig. 1: (a), (b), and (c) Numerical simulation of the intensity corresponding to Debye–Wolf (or diffraction) integrals I_{00}, I_{01}, and I_{02} at $z = 2\,\mu$m away from the focus for a refractive index (RI) of 1.814, respectively. (d) Comparison of the strength of the Debye–Wolf integrals at the on-axis (beam centre) and off-axis positions in the intensity distribution.

2 Numerical Simulation

Our simulations are performed for the tight focusing of an input circularly polarized Gaussian beam by a high NA objective lens into a stratified medium, as described in the main manuscript (see section

5 and Fig. 5). In the focal plane (or near the focal plane), the electric field exhibits components not only along the transverse direction but also along the longitudinal direction, due to the transverse boundary conditions. As mentioned in the manuscript, the electric field intensity at the center of the beam profile appears as bright spots because the zero-order Bessel function (J_0, embedded in I_{00}), has a non-vanishing value at the origin (on-axis). However, the intensity corresponding to the longitudinal component of the electric field (E_z) is primarily concentrated at off-axis positions due to the first-order Bessel function (J_1), embedded in the I_{01} coefficient. In Figs. 1 (a), (b), and (c), we show the distribution of the squared moduli of I_{00}, I_{01}, and I_{02}, respectively. However, in Fig. 1 (d), we compare the strength of the Debye–Wolf integrals at the on-axis (beam center) and off-axis positions. The maximum value of the squared modulus of I_{00} occurs at both the on-axis (beam center) and off-axis positions, with a value around 2700 (arb. units). In contrast, the maximum value of the squared modulus of I_{01} primarily occurs at off-axis positions, with a value around 230 (arb. units), while the squared modulus of I_{02} is negligible.

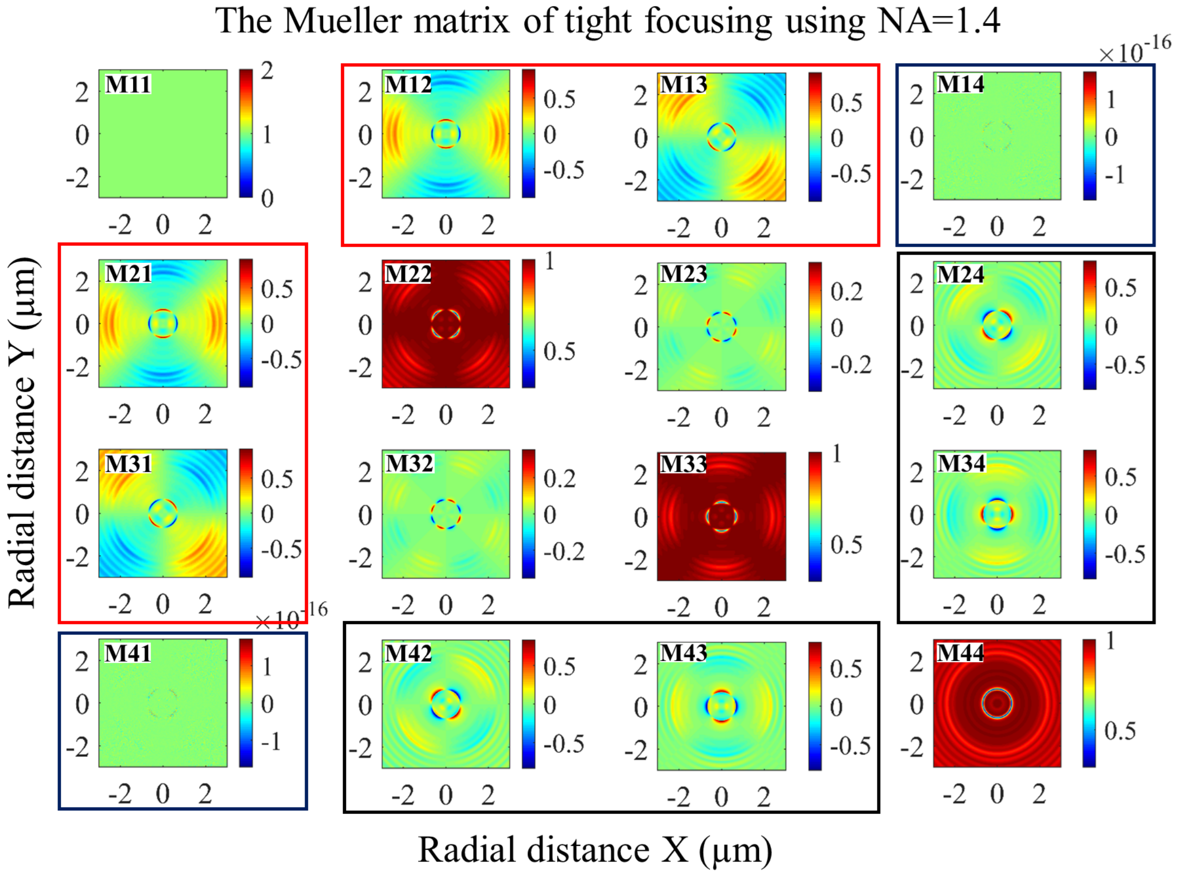

Fig. 2: Numerically computed 4x4 Mueller matrix for the tight focusing of a Gaussian beam. The signature of the linear diattenuator is indicated by elements $M_{12} = M_{21} \propto \cos(2\psi)$ and $M_{13} = M_{31} \propto \sin(2\psi)$ (marked by red boxes). The linear retarder is indicated by elements M_{24} = -$M_{42} \propto -\sin(\delta)\sin(2\psi)$ and M_{34} = -$M_{43} \propto \sin(\delta)\cos(2\psi)$ (marked by black boxes). The elements M_{14} and $M_{41} = 0$, which represent circular diattenuation (or circular anisotropy), are highlighted with blue boxes. The elements M_{23} and M_{32} illustrate the gradient of the geometric phase and are $\propto \cos(2\psi)\sin(2\psi)(1-\cos\delta)$. The diagonal element M_{11} represents the total intensity of the focused field, while the remaining elements M_{22}, M_{33}, and M_{44} describe the depolarization parameters.

2.1 Mueller matrix of tight focusing

To analyze the SOI effect using conventional polarization parameters such as diattenuation (d) and retardance (δ), we derive the Mueller matrix corresponding to the Jones matrix of tight focusing. Diattenuation represents the differential attenuation of orthogonal polarizations, while retardance accounts for the phase anisotropy, i.e., the phase difference introduced between orthogonal polarization states. These polarization effects relate to the amplitude and phase components of the focused field, respectively. The Mueller matrix corresponding to the 2x2 Jones matrix J (extracted from the first two rows and columns of the 3x3 Jones matrix in Eq. 4) can be derived using the standard formula $M = \mathcal{A} \cdot (J \otimes J^*) \cdot \mathcal{A}^{-1}$. Here, J is the 2x2 Jones matrix for tight focusing, and $\mathcal{A}$ is a 4x4 matrix defined as [4, 5]:

$$\mathcal{A} = \begin{pmatrix} 1 & 0 & 0 & 1 \\ 1 & 0 & 0 & -1 \\ 0 & 1 & 1 & 0 \\ 0 & -i & i & 0 \end{pmatrix}; \quad J = \begin{bmatrix} I_{00} + I_{02}\cos 2\psi & I_{02}\sin 2\psi \\ I_{02}\sin 2\psi & I_{00} - I_{02}\cos 2\psi \end{bmatrix},$$

The resulting matrix is a diattenuating retarder Mueller matrix, $M_{TF}(d_{TF}, \delta_{TF}, \psi)$, characterized by diattenuation d_{TF}, retardance δ_{TF}, and azimuthal angle ψ, which represents the orientation of the axes of the diattenuating retarder. The subscript TF denotes the tight focusing. In Fig. 2, we present the numerically computed 4x4 Mueller matrix for an azimuthal diattenuating retarder. The matrix elements that represent the linear diattenuator are M_{12} and M_{21}, which are proportional to $\cos(2\psi)$, and M_{13} and M_{31}, which are proportional to $\sin(2\psi)$; these are marked with red boxes. The linear retarder is characterized by elements M_{24} and M_{42}, which are proportional to $-\sin(\delta)\sin(2\psi)$, and M_{34} and M_{43}, which are proportional to $\sin(\delta)\cos(2\psi)$; these elements are highlighted with black boxes. Elements M_{14} and M_{41}, which are highlighted with blue boxes, are equal to zero and represent circular diattenuation (or circular anisotropy). Additionally, elements M_{23} and M_{32} illustrate the gradient of the geometric phase, being proportional to $\cos(2\psi)\sin(2\psi)(1 - \cos\delta)$. The diagonal element M_{11} represents the total intensity of the focused field, while the other diagonal elements M_{22}, M_{33}, and M_{44} denote the depolarization parameters.

2.2 Mueller matrix of customized LC particles

The general Mueller matrix elements (M_{ij}, where i represents the row and j the column, with $i, j = 1, 2, 3, 4$) corresponding to a diattenuating retarder are given as follows[5–9]:

$$\begin{aligned}
&M_{11} = 1; \quad M_{12} = M_{21} = d\cos 2\psi; \quad M_{13} = M_{31} = d\sin 2\psi; \\
&M_{14} = M_{41} = 0; \quad M_{22} = \cos^2 2\psi + x\cos\delta\sin^2 2\psi; \\
&M_{23} = M_{32} = \sin 2\psi\cos 2\psi - x\cos\delta\sin 2\psi\cos 2\psi; \\
&M_{24} = -M_{42} = -x\sin\delta\sin 2\psi; \\
&M_{33} = \sin^2 2\psi + x\cos\delta\cos^2 2\psi; \\
&M_{34} = -M_{43} = x\sin\delta\cos 2\psi; \\
&M_{44} = x\cos\delta; \quad x = \left|\sqrt{1-d^2}\right|.
\end{aligned} \tag{8}$$

Here, d represents the diattenuation, δ is the retardance, and the azimuthal angle ψ corresponds to the orientation of the axes of the diattenuating retarder. We first quantified the polarization parameters (diattenuation d and retardance δ) of the LC particle using the Lu-Chipman (or polar) decomposition method [4, 10, 11], with detailed information provided in Section 6.3 of the Experimental Methods in

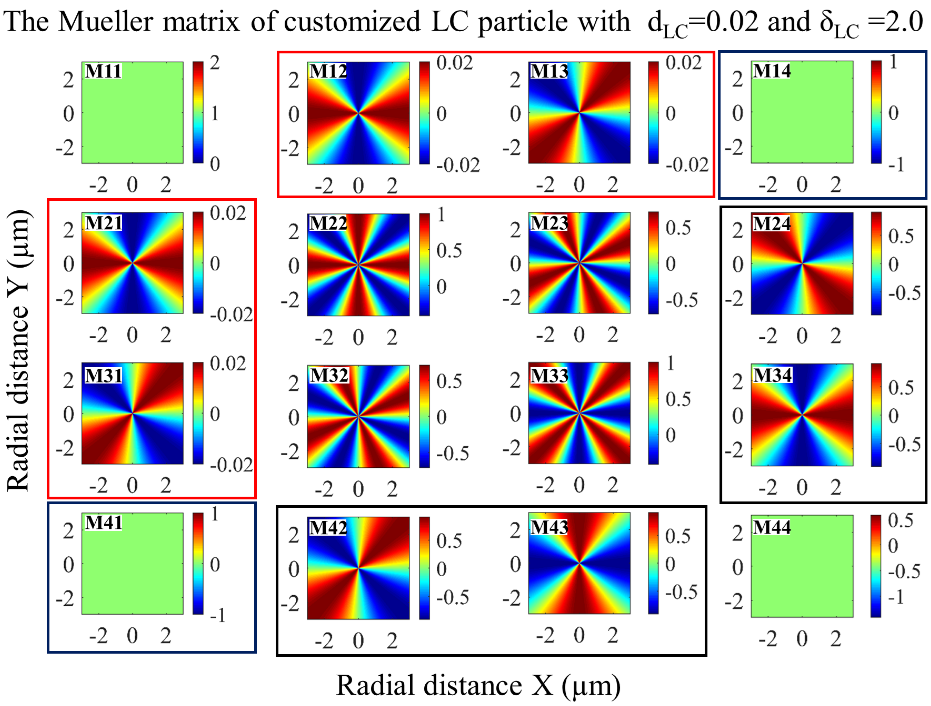

Fig. 3: Mueller matrix of a customized LC particle with linear diattenuation $d_{LC} = 0.02$ and linear retardance $\delta_{LC} = 2.0$. The signature of the linear diattenuator is indicated by elements M_{12}, M_{13}, M_{21}, and M_{31} (red boxes). The linear retarder is represented by elements M_{24}, M_{34}, M_{42}, and M_{43} (black boxes). Elements M_{14} and M_{41}, highlighted in blue, correspond to circular diattenuation, but their values are zero for LC particles. The element M_{44} remains constant. Similar Mueller matrix features are also observed for $\delta_{LC} = 1.2$ and $\delta_{LC} = 1.57$.

the main manuscript (see Fig. 7). The experimentally observed values of $d = 0.02$ and $\delta = 2.0$ were then substituted into Eq. 8, allowing us to numerically compute the Mueller matrix of the customized LC particle, $M_{LC}(d_{LC}, \delta_{LC}, \psi)$, as shown in Fig. 3. Experimentally, we collected multiple sets of data and observed that our sample exhibited a relatively constant diattenuation value of around $d = 0.02$. However, the linear retardance (δ_{LC}) values varied between approximately 0.5 and 3. We categorized these into three distinct groups: $\delta_{LC} < \pi/2$, $\delta_{LC} = \pi/2$, and $\delta_{LC} > \pi/2$. Here, we present the results corresponding to $\delta_{LC} > \pi/2$ in Fig. 3, noting that for the other two cases ($\delta_{LC} < \pi/2$ and $\delta_{LC} = \pi/2$), the distribution of the Mueller matrix elements (M_{ij}) remains similar, with differences only in the maximum and minimum values.

2.3 Mueller matrix of composite effect

The tight focusing of the beam and the bipolar variation of the anisotropy axis of the LC particles together create a composite effect. To model the composite effects, we calculated the resultant Mueller matrix $M_{res}(d, \delta, \psi)$, which is the sequential product of the Mueller matrix of tight focusing, $M_{TF}(d_{TF}, \delta_{TF}, \psi)$, and the Mueller matrix of the customized LC particle, $M_{LC}(d_{LC}, \delta_{LC}, \psi)$. This represents two successive polarization-transforming events, such that $M_{res}(d, \delta, \psi) = M_{TF}(d_{TF}, \delta_{TF}, \psi) \cdot M_{LC}(d_{LC}, \delta_{LC}, \psi)$. Here, we show the results for the case of $\delta_{LC} > \pi/2$ in Fig. 4. For the cases of $\delta < \pi/2$ and $\delta_{LC} = \pi/2$, the distribution of the resultant Mueller matrix elements (M_{ij}) remains similar to that of the earlier case ($\delta_{LC} > \pi/2$), with differences only in the maximum and minimum values. Now, we then calculate the resulting Stokes vector using the equation $S^{out} = M_{res}\ S^{in}$, where M_{res} is the 4x4 Mueller matrix that represents the combined effect, and S_{in} and S_{out} are the Stokes vectors for the input and output fields, respectively.

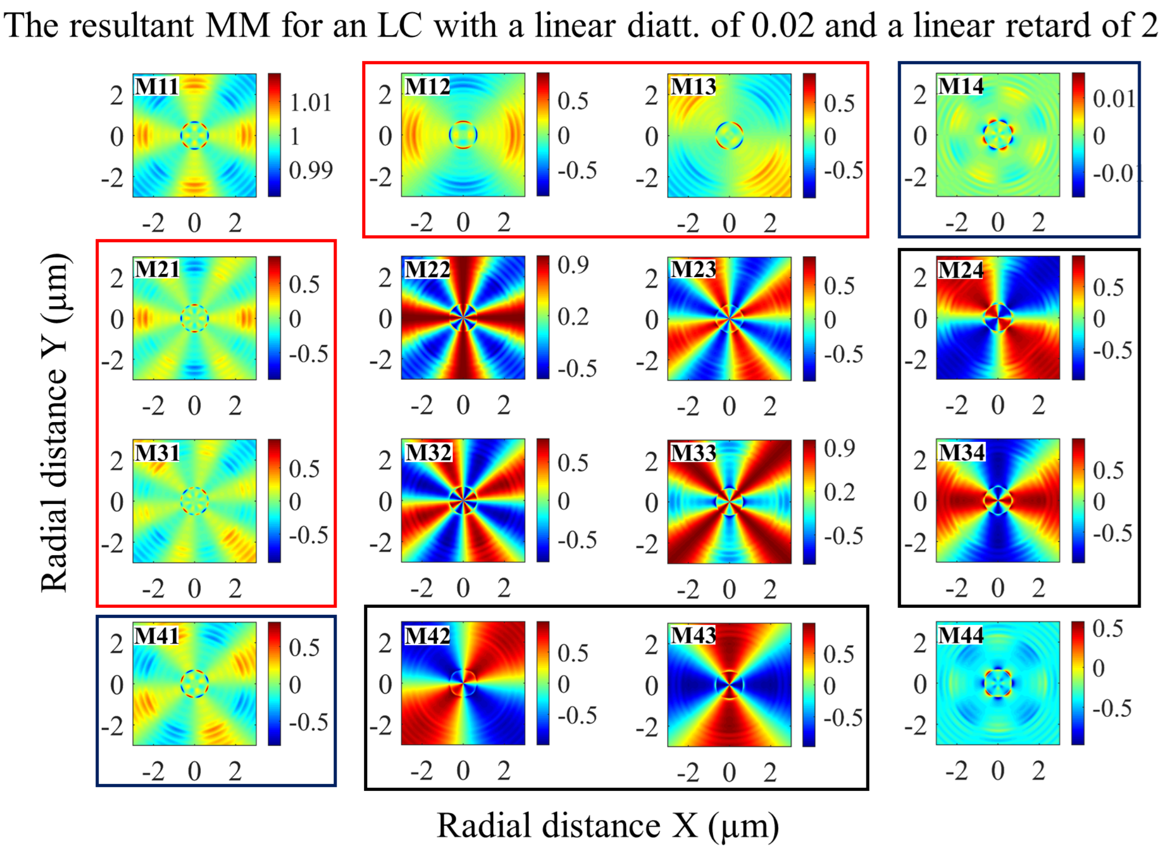

Fig. 4: Numerically computed 4×4 resultant Mueller matrix for the tight focusing of a Gaussian beam on an LC particle with linear diattenuation $d_{LC} = 0.02$ and linear retardance $\delta_{LC} = 2.0 > \pi/2$. The signature of the linear diattenuator is indicated by the elements M_{12}, M_{13}, M_{21}, and M_{31} (red boxes). The linear retarder is indicated by the elements M_{24}, M_{34}, M_{42}, and M_{43} (black boxes). The elements M_{14} and M_{41}, which describe the Spin Hall Effect (SHE)—i.e., the difference between the RCP and LCP components of the electric field in the focal plane—are highlighted in blue. Note that the resultant Mueller matrices for $\delta_{LC} = 1.2 < \pi/2$ and $\delta_{LC} = 1.57 \sim \pi/2$ exhibit similar features.

2.4 Resultant Stokes Vector

Based on our experimental results, we computed the resultant Stokes vector elements $S0$, $S1$, $S2$, and $S3$ for input left-circularly polarized (LCP) light ($S^{in}_{LCP} = [1\ 0\ 0\ -1]^T$) and right-circularly polarized (RCP) light ($S^{in}_{RCP} = [1\ 0\ 0\ 1]^T$). These were obtained using the resultant Mueller matrix M_{res}, which captures the composite effect of tight focusing of a Gaussian beam on an LC particle, according to $S^{out} = M_{res}\, S^{in}$. In Fig. 5, we show the resultant Stokes vectors for fixed linear diattenuation ($d_{LC} = 0.02$) and linear retardance $\delta_{LC} > \pi/2$. The overall distribution is similar to the cases $\delta_{LC} < \pi/2$ and $\delta_{LC} = \pi/2$, differing only in the maximum and minimum values. Fig. 5 presents a comprehensive visualization of the Stokes parameters along with the resulting indirect helicities of the emerging (or scattering) field. Panels (a) and (e) show the $S0$ parameter, corresponding to the normalized total intensity. The variations in the linearly polarized components are illustrated in panels (b), (c), (f), and (g), where the $S1$ parameter denotes the difference between the horizontally (H) and vertically (V) polarized components, and the $S2$ parameter indicates the difference between the diagonally (D) and anti-diagonally (A) polarized components. Panels (d) and (h) show the $S3$ component of the Stokes parameters, which represents the spatial distribution of the indirect helicities generated in the system.

Since the $S3$ component of the Stokes vector is equivalent to the longitudinal spin angular momentum (LSAM) density, it is of particular interest to examine its distribution, as it fully accounts for the generation of indirect helicities. In Figs. 6(a)–(c), we plot the $S3$ component of the resultant

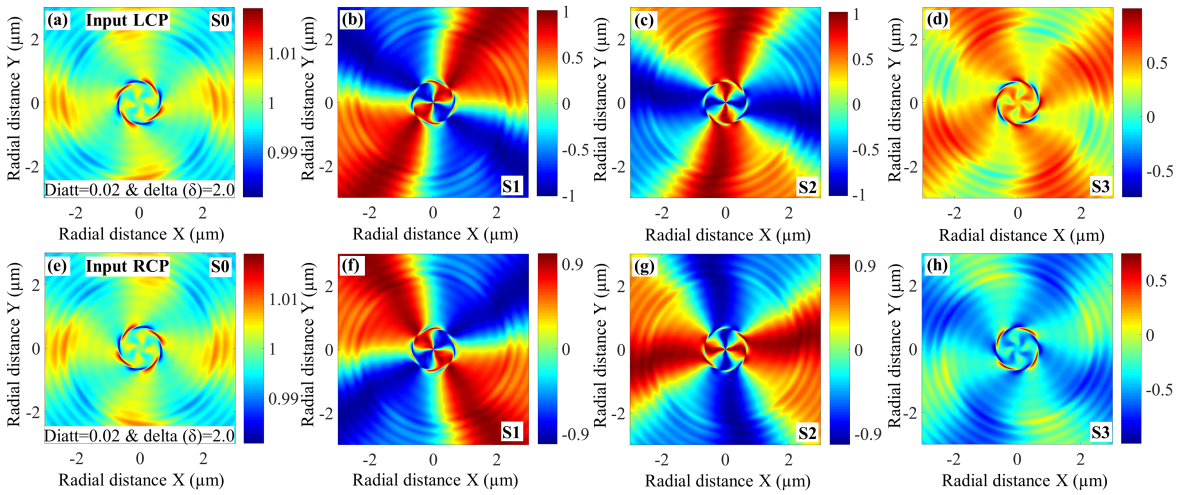

Fig. 5: The resultant Stokes vector elements for tightly focused circularly polarized light on an LC particle with a fixed linear diattenuation value of 0.02 and a linear retardance of 2.0. (a)-(d) show results for left circularly polarized light, while (e)-(h) show results for right circularly polarized light. (a) and (e) depict the $S0$ component, representing the normalized total intensity. (b), (c), (f), and (g) display the $S1$ and $S2$ components, which describe the differences in the linearly polarized components of the emerging field. (d) and (h) illustrate the $S3$ component, which is proportional to the longitudinal component of spin angular momentum (LSAM) density, highlighting the spatial separation between $\sigma = +1$ and $\sigma = -1$ helicity of the scattered field from the primary LC particle.

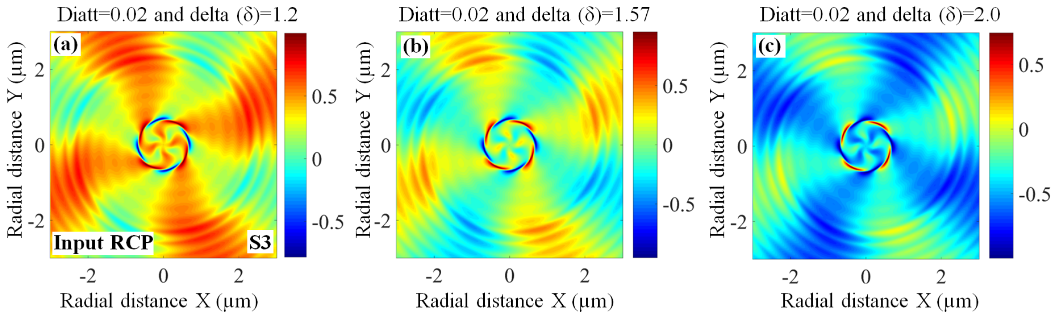

Fig. 6: The resultant $S3$ component of Stokes vector elements for tightly focused circularly polarized light on an LC particle at a fixed linear diattenuation value of 0.02 and different linear retardance values. (a), (b), and (c) show the results for right circularly polarized (RCP) light at 1.2, 1.57, and 2.0, respectively.

Stokes vector for an input RCP Gaussian beam in the three regimes: $\delta_{LC} < \pi/2$, $\delta_{LC} = \pi/2$, and $\delta_{LC} > \pi/2$. The corresponding results for an input LCP beam are already presented in the main manuscript (Fig. 2(a)–(c)). By convention, positive (σ_+) and negative (σ_-) values of $S3$ represent clockwise and counterclockwise spinning of the LC particle about the z-axis, respectively. The simulated results in Figs. 6 (a)–(c) corroborate our theoretical predictions: when $\delta_{LC} < \pi/2$ ($\delta_{LC} = 1.2$), the same-helicity component dominates at the off-axis positions (Fig. 6 (a)), so both central and off-axis particles spin in the same direction, as predicted by Eq.(3) of the manuscript. When $\delta_{LC} = \pi/2$, the coefficients of both helicities (σ_+ and σ_-) are equal, yielding spatially separated regions of σ_+ and σ_- (Fig. 6 (b)), such that the spin direction of an off-axis particle depends on its location (clockwise in σ_+ regions, counterclockwise in σ_- regions). Finally, when $\delta_{LC} > \pi/2$ ($\delta_{LC} = 2$), the opposite-helicity component dominates (Fig. 6 (c)), and the orbiting secondary particles spin opposite to the centrally trapped primary one. These numerical simulations validate the emergence of indirect helicities driven by extrinsic SOI effects, as described by Eq. (3) of the manuscript.

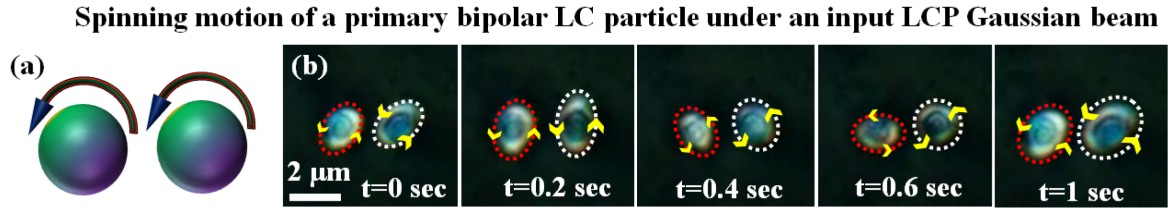

Fig. 7: (a) Cartoon representation of spatially separated spinning bipolar LC particles illustrating direct helicity transfer. (b) Time-lapsed frames from Video 5 showing spatially separated bipolar LC particles (within red and white dotted ellipses) spinning counterclockwise, confirming direct helicity transfer. (See Video 5 in the Supplemental Material).

3 Experimental results

3.1 Effect of Direct Helicity on Spatially Separated Particles

The schematic in Fig. 7(a) illustrates the spinning motion of primary particles driven by the direct helicity of light. In this case, we show the results of two spatially separated (or independent) bipolar micro LC particle spinning due to the LSAM present in a tightly focused circularly polarized Gaussian beam. To obtain quantitative proof of the effects of the LSAM (or direct helicity), we utilized a cross-polarization scheme, as detailed in the Experimental Method section 6 of the main manuscript. As the LC particles spin due to helicity transfer, the intensity pattern across their surface continuously changes throughout the time-lapse images. Thus, the rotation in the xy-plane (i.e., about the beam propagation z-axis) implies that the nature of the SAM is longitudinal. In Fig. 7(b) (time-lapsed images from 'Video 5'), the LC particles within the red and white dotted ellipses spin counterclockwise (indicated by the small yellow arrows on the dotted ellipses) for input LCP light. Both LC particles are trapped in the spherically aberrated intensity profile of a tightly focused circularly polarized Gaussian beam under the mismatched condition of RI 1.814, as shown in Fig. 5 (c) of the main manuscript. Due to direct helicity ($\sigma = -1$) transfer from the input beam, the spatially separated LC particles spin in the same counterclockwise direction (see 'Video 5' in the online Supplemental Material).

3.2 Effect of Indirect Helicity on Radial and Bipolar LC Particles

The spinning primary LC particle at the beam center (on-axis) induces a fluid (or spin) flow in its surroundings, causing smaller LC particles to orbit in the direction of this flow. An LCP-input Gaussian beam generates a counterclockwise fluid flow, while an RCP-input Gaussian beam produces a clockwise flow. In Fig. 8(b) (time-lapsed images from Video 6), the orbiting LC particle does not spin. This occurs because the off-axis secondary LC particle exhibits a spherically symmetric director configuration, which blocks indirect helicity transfer; consequently, the orbiting radial LC particle cannot spin on its axis (see Video 6 in the online Supplemental Material). In contrast, Fig. 8(d) (time-lapsed images from Video 7) shows that the central particle, highlighted by the red dotted circle, spins counterclockwise due to direct helicity transfer, while off-axis LC particles orbit under the induced fluid flow, as indicated by the yellow arrow lines. Simultaneously, the orbiting LC particles also rotate about their own axes due to indirect helicity transfer mediated by the centrally trapped primary particle. In this case, both the central and orbiting LC particles are bipolar, with asymmetrically oriented anisotropic directors; thus, the bipolar configuration of the LC directors is spin-responsive. In contrast, a radial configuration of LC directors is spin-irresponsive. The spinning direction of the secondary particles depends entirely on the linear birefringence of the centrally trapped primary LC particle (see Video 7 in the Supplemental Material). Further details are discussed in the main text.

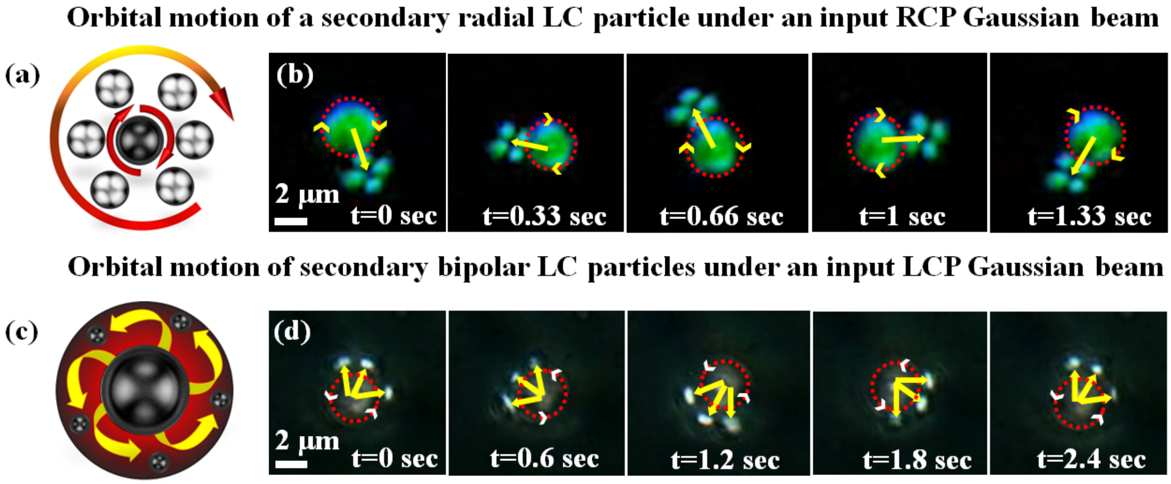

Fig. 8: (a) and (c) Cartoon representations of the orbital motion of secondary radial and bipolar LC particles, respectively. (b) Time-lapsed frames from Video 6 showing secondary radial LC particles (indicated by yellow arrow line) orbiting at off-axis positions, driven by the fluid flow from a primary bipolar LC particle (within red dotted circle) at the beam center under an input RCP Gaussian beam. The orbiting LC particles do not spin due to their radial director symmetry. (d) Time-lapsed frames from Video 7 showing secondary bipolar LC particles orbiting (indicated by yellow arrow lines) and additionally spinning on their axes due to indirect helicity transfer, under an input LCP Gaussian beam. The bipolar LC particles have an asymmetric director axis. Both videos were recorded under cross-polarization (see Video 6 and Video 7 in the Supplemental Material).

4 Experimental Methods

We employ a conventional optical tweezers setup, consisting of an inverted microscope (Carl Zeiss Axiovert.A1) with a 100X oil-immersion objective (Zeiss, NA 1.4). To determine the Mueller matrix of LC particles, we integrate a polarization state generator (PSG) at the microscope's input and a polarization state analyzer (PSA) at the output. The PSG, which includes a polarizer and a quarter-wave plate, generates six distinct linear and circular polarization states. Collimated white light from the microscope's built-in illumination source (a 100W halogen lamp) passes through the PSG, featuring a rotatable linear polarizer and a quarter-wave plate optimized for 671 nm. This light is then focused onto the birefringent LC sample, and the scattered light is collected by the microscope objective (Carl Zeiss Axio-vert.A1, NA 1.4). The PSA, comprising a quarter-wave plate and a linear polarizer, then analyzes the polarization state of the scattered light. For each polarization state generated by the PSG, six measurements are taken with the PSA, yielding a total of 36 polarization-resolved measurements. These measurements are used to construct the 4x4 Mueller matrix as [5, 11]

HH+HV+VH+VV	HH+HV−VH−VV	PH+PV−MH−MV	RH+RV−LH−LV
HH−HV+VH−VV	HH−HV−VH+VV	PH−PV−MH+MV	RH−RV−LH+LV
HP+VP−HM−VM	HP−VP−HM+VM	PP−PM−MP+MM	RP−RM−LP+LM
HR+VR−HL−VL	HR−VR−HL+VL	PR−PL−MR+ML	RR−RL−LR+LL

Tab. 1: Scheme for construction of 4X4 Mueller matrix using 36 polarization-resolved projective measurements. Here, the first letter represents the input polarization state, and the second letter stands for the analyzer or the projected polarization state. The states are defined as $I_H(horizontal)$, $I_V(vertical)$, $I_P(+45\,\deg)$, $I_M(-45\,\deg)$, I_L left circular polarized (LCP), $I_R(RCP)$

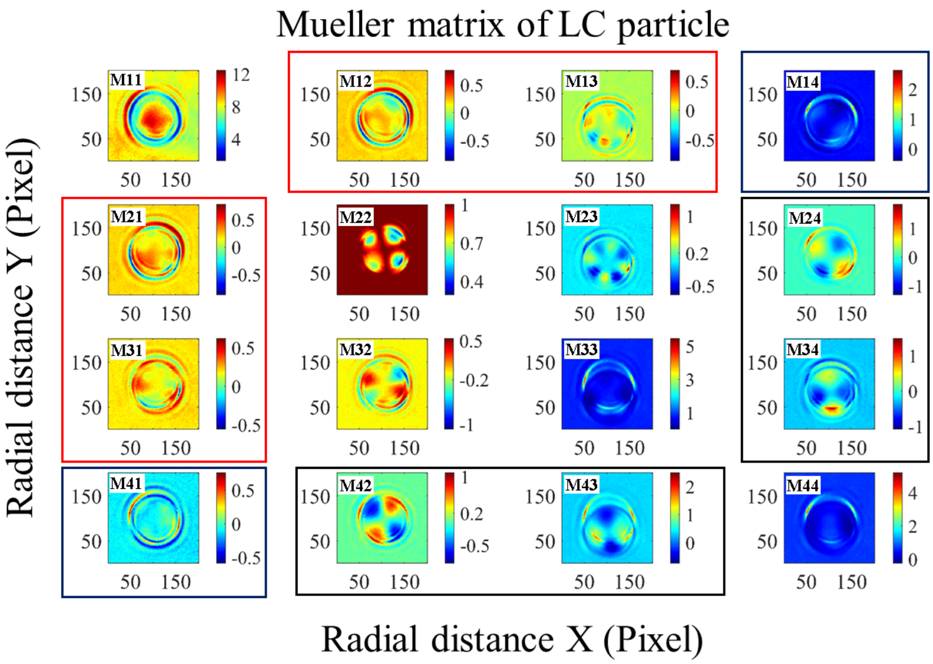

Fig. 9: Experimentally measured Mueller matrix of an LC particle. Linear diattenuation is identified by elements M_{12}, M_{13}, M_{21}, and M_{31} (red boxes). Linear retardance is represented by elements M_{24}, M_{34}, M_{42}, and M_{43} (black boxes). Elements M_{14} and M_{41} correspond to circular diattenuation (blue boxes)

Using the Lu–Chipman (polar) decomposition method [4, 10, 11], we quantified the polarization parameters of the LC particles, namely linear diattenuation (d_{LC}) and linear retardance (δ_{LC}), as illustrated in the main manuscript. From multiple experimental datasets, we observed that the diattenuation remained relatively constant at $d_{LC} \approx 0.02$. In contrast, the retardance varied between 0.5 and 3, which we categorized into three regimes: $\delta_{LC} < \pi/2$, $\delta_{LC} \approx \pi/2$, and $\delta_{LC} > \pi/2$. In Sec. 2.2, we used these polarization parameters to construct customized Mueller matrices of LC particles. Here, in Fig. 9, we present the experimentally measured Mueller matrix of an actual LC particle. The full matrix elements were obtained using the projective measurement scheme described in Table 1.

5 Video Captions

- **Video 1** and **Video 2:** The simultaneous spinning and orbiting (planetary-like) motion of primary and secondary LC particles for input LCP and RCP light, respectively. The on-axis trapped particle ($\delta_{LC} > \pi/2$) spins counterclockwise and clockwise due to direct helicity transfer, respectively, while the off-axis trapped particle orbits due to fluid flow and spins with the opposite helicity (clockwise and counterclockwise) as the input helicity due to indirect helicity transfer.
- **Video 3:** The simultaneous spinning and orbiting (planetary-like) motion of primary and secondary LC particles for input LCP light. The on-axis trapped particle ($\delta_{LC} < \pi/2$) spins counterclockwise due to direct helicity transfer, while the off-axis trapped particle orbits due to fluid flow and spins with the same helicity (counterclockwise) as the input helicity due to indirect helicity transfer.
- **Video 4:** Simultaneous spinning motion of spatially resolved secondary LC particles for input RCP light. The on-axis trapped large particle ($\delta_{LC} \sim \pi/2$) at the center of the cluster of trapped particles spins clockwise due to direct helicity transfer, while the off-axis trapped particles spin

with both the same and opposite helicity relative to the input helicity due to indirect helicity transfer.

- **Video 5:** Anti-clockwise spinning of liquid crystal (LC) micro-particles trapped in a spatially spherical aberrated region for mismatch RI 1.814 of the coverslip under the tight focusing of a left circularly polarized (LCP) Gaussian beam. This motion occurs due to the primary action of input helicity. This video was recorded using a cross-polarization mechanism for the input LCP light during the experiment.
- **Video 6:** The spinning bipolar LC at the beam center (on-axis) induces a fluid flow in its surroundings, causing the radial LC particle to orbit in the direction of this flow. The orbiting secondary radial LC particle does not spin at the off-axis position of the trap due to the spherically symmetric distribution of its anisotropy axis. This video was recorded using a cross-polarization mechanism for the input RCP light during the experiment. That is why the four polarization lobes appear in the radial LC particle.
- **Video 7:** The spinning LC particle at the beam center (on-axis) induces a fluid (or spin) flow in its surroundings, causing smaller LC particles to orbit in the direction of this flow. Both the spinning and orbiting LC particles are bipolar, with asymmetrically oriented anisotropic directors. Therefore, the orbiting LC motors are also spinning. This video was recorded using a cross-polarization mechanism for the input LCP light during the experiment.

References

[1] L. Novotny and B. Hecht, *Principles of Nano-Optics*, Cambridge University Press, Cambridge, 2012.

[2] B. Roy, N. Ghosh, S. D. Gupta, P. K. Panigrahi, S. Roy, and A. Banerjee, "Controlled transportation of mesoscopic particles by enhanced spin-orbit interaction of light in an optical trap," *Phys. Rev. A*, vol. 87, no. 4, p. 043823, 2013.

[3] B. Richards and E. Wolf, "Electromagnetic diffraction in optical systems, II. Structure of the image field in an aplanatic system," *Proc. R. Soc. Lond. A*, vol. 253, no. 1274, pp. 358–379, 1959.

[4] S.-Y. Lu, *An Interpretation of Polarization Matrices*, The University of Alabama in Huntsville, Huntsville, AL, 1995.

[5] S. D. Gupta, N. Ghosh, and A. Banerjee, *Wave Optics: Basic Concepts and Contemporary Trends*, CRC Press, Boca Raton, FL, 2015.

[6] J. N. Hilfiker, C. M. Herzinger, T. Wagner, A. Marino, G. Delgais, and G. Abbate, "Mueller-matrix characterization of liquid crystals," *Thin Solid Films*, vol. 455, pp. 591–595, 2004.

[7] A. Zaidi, N. A. Rubin, M. L. Meretska, L. W. Li, A. H. Dorrah, J.-S. Park, and F. Capasso, "Metasurface-enabled single-shot and complete Mueller matrix imaging," *Nat. Photonics*, pp. 1–9, 2024.

[8] J. Soni, S. Ghosh, S. Mansha, A. Kumar, S. D. Gupta, A. Banerjee, and N. Ghosh, "Enhancing spin–orbit interaction of light by plasmonic nanostructures," *Opt. Lett.*, vol. 38, no. 10, pp. 1748–1750, 2013.

[9] S.-Y. Lu and R. A. Chipman, "Interpretation of Mueller matrices based on polar decomposition," *J. Opt. Soc. Am. A*, vol. 13, no. 5, pp. 1106–1113, 1996.

[10] Z.-F. Xing, "On the deterministic and non-deterministic Mueller matrix," *J. Mod. Opt.*, vol. 39, no. 3, pp. 461–484, 1992.

[11] J. K. Nayak, H. Suchiang, S. K. Ray, S. Guchhait, A. Banerjee, S. D. Gupta, and N. Ghosh, "Spin-direction-spin coupling of quasiguided modes in plasmonic crystals," *Phys. Rev. Lett.*, vol. 131, no. 19, p. 193803, 2023.
