# Supplementary figures and images for: Harvesting the Spin–Orbit Interaction of Light to Generate Helicity‐Dependent Complex Rotational Motion in Optically Trapped Mesoscopic Matter
Source: Nanophotonics. 2026 Feb 24;15(5):e70034. doi: 10.1002/nap2.70034 (PMC12965037; doi:10.1002/nap2.70034)

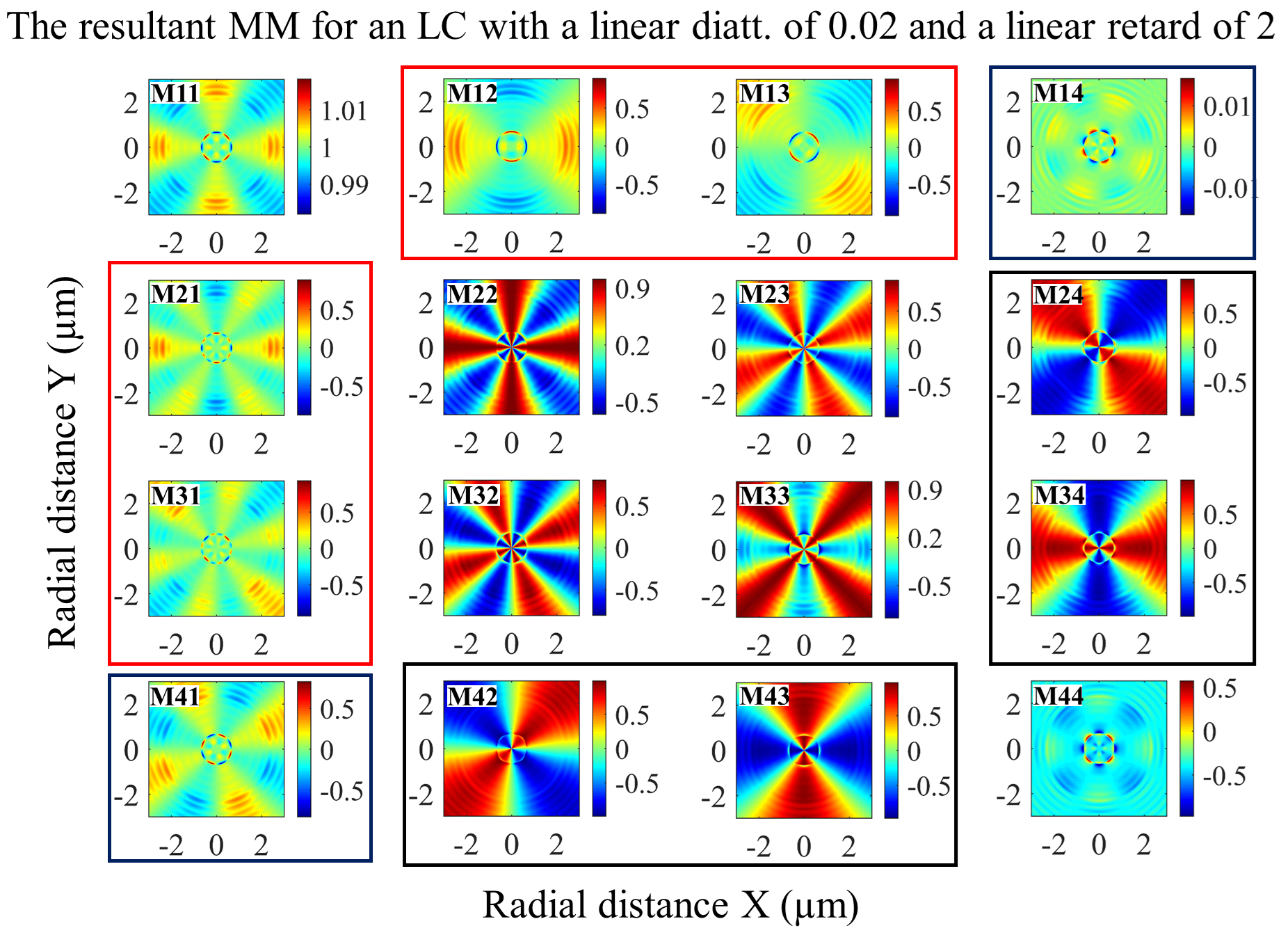

Supplement: Supplementary file 1 — Supporting Information S1 [file NAP2-15-e70034-s001.zip › Fig_4_SM.png]

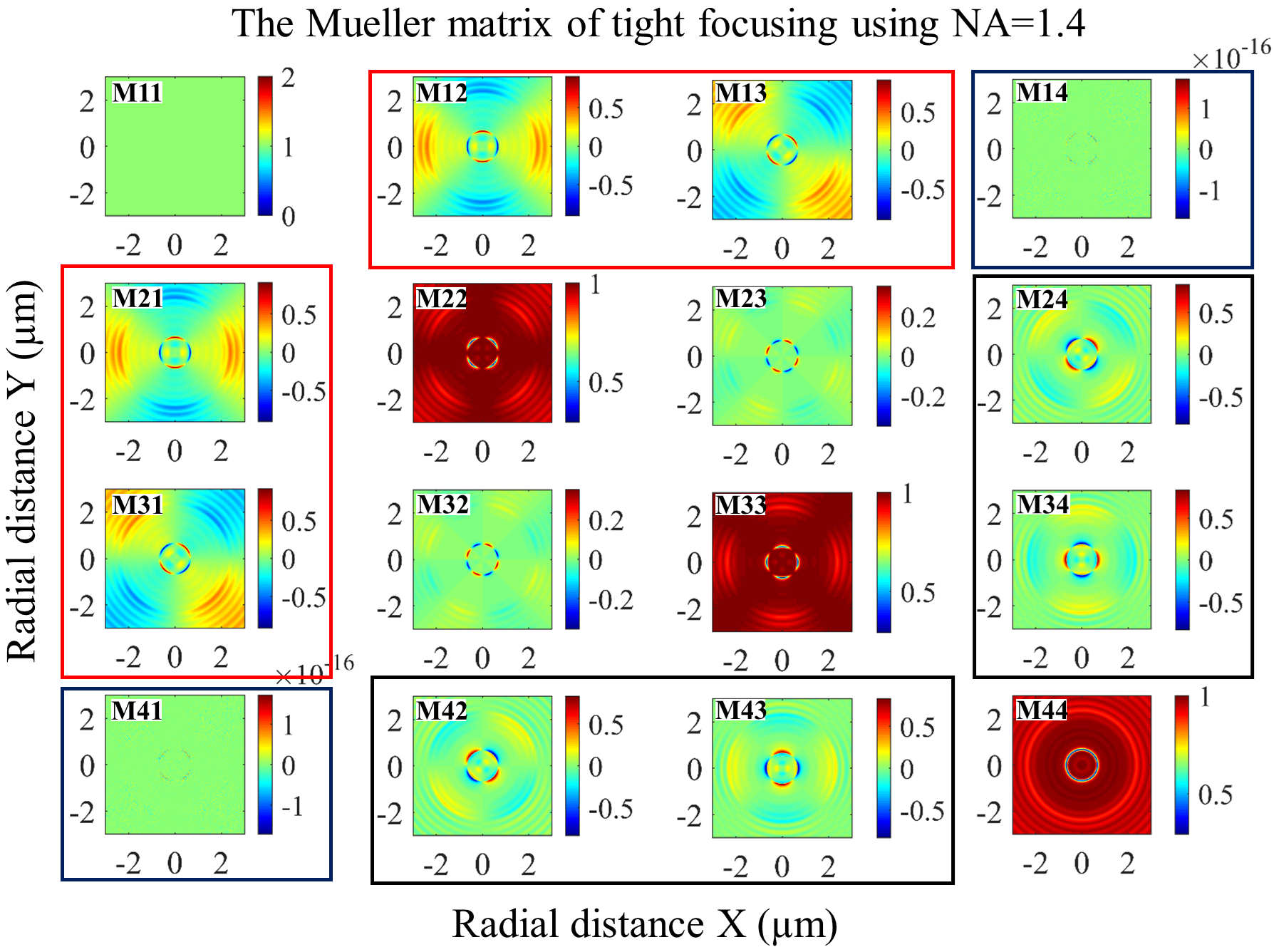

Supplement: Supplementary file 1 — Supporting Information S1 [file NAP2-15-e70034-s001.zip › Fig_2_SM.png]

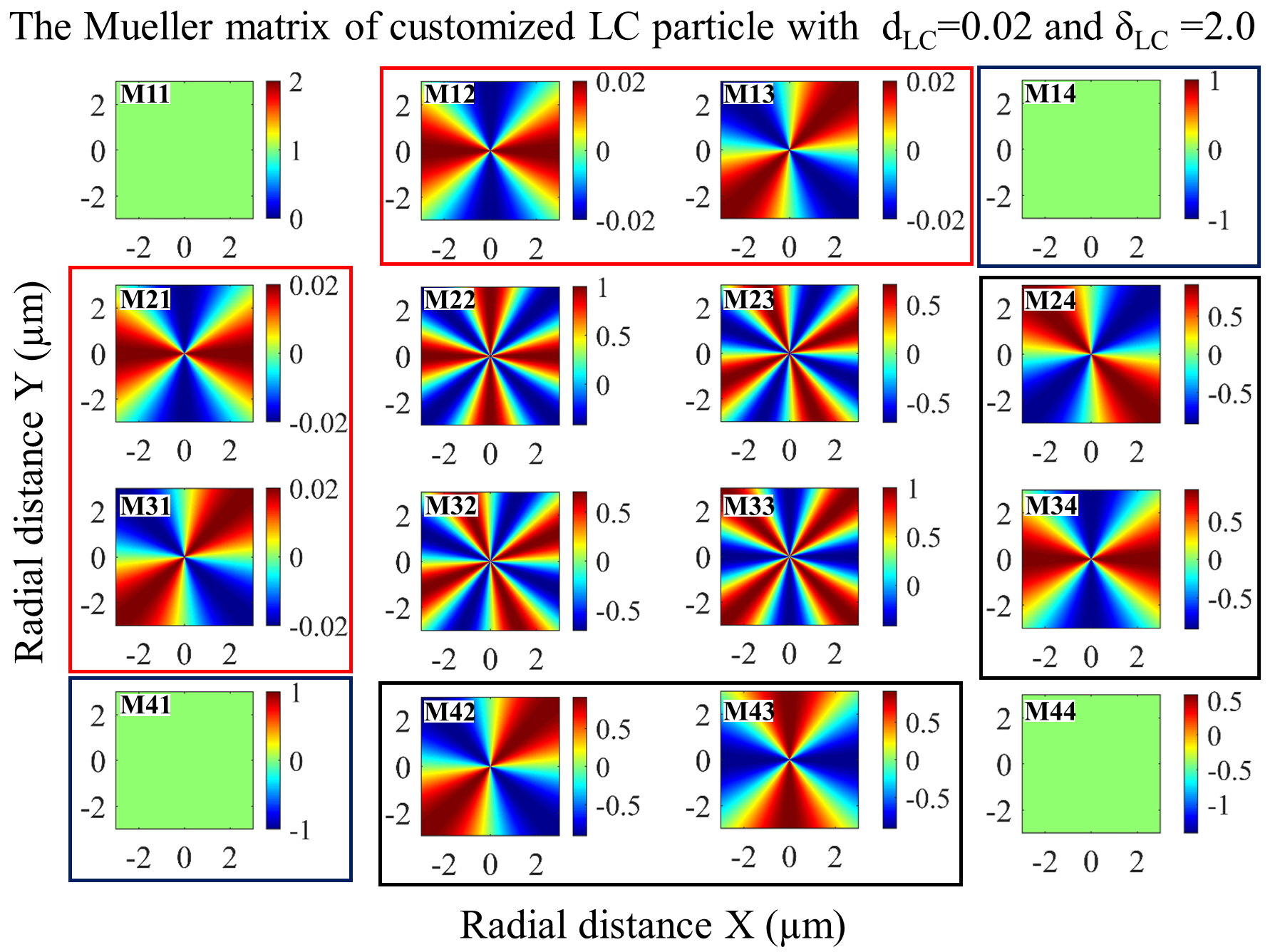

Supplement: Supplementary file 1 — Supporting Information S1 [file NAP2-15-e70034-s001.zip › Fig_3_SM.png]

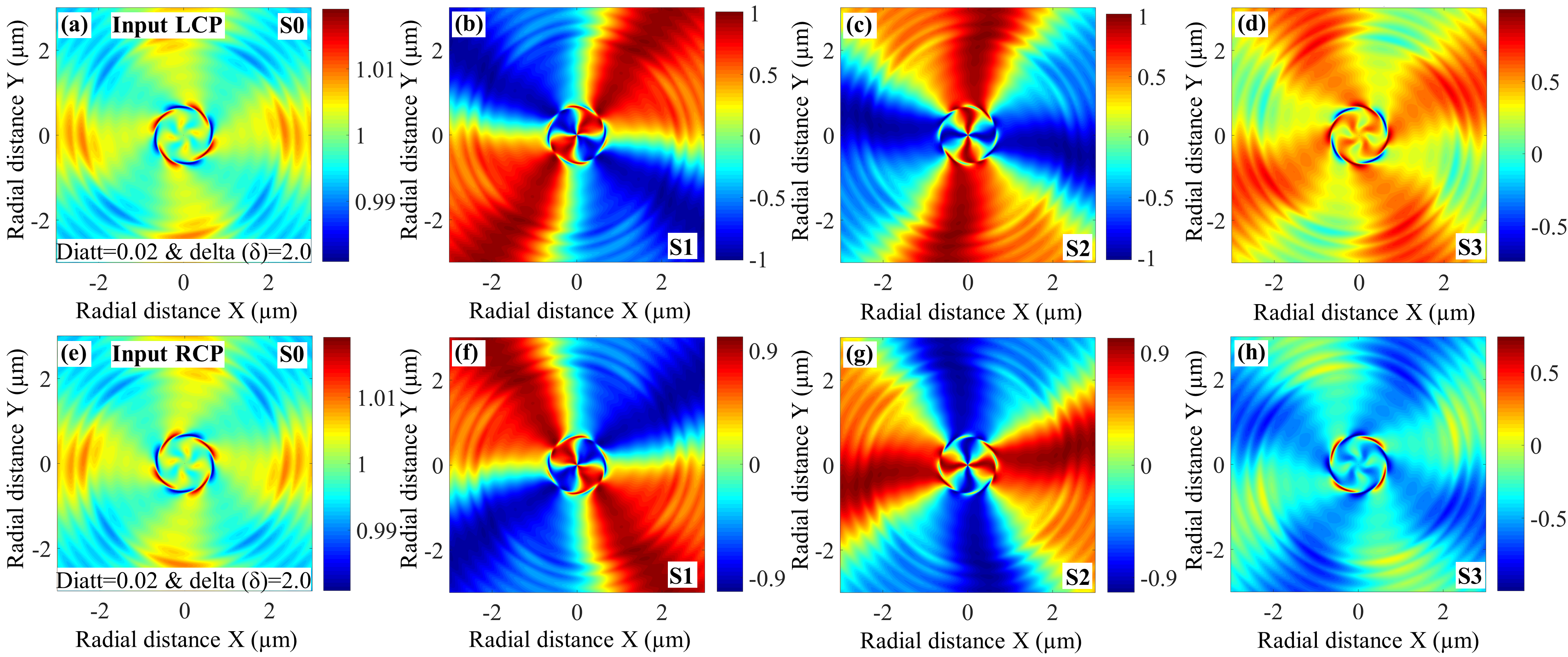

Supplement: Supplementary file 1 — Supporting Information S1 [file NAP2-15-e70034-s001.zip › Fig_5_SM.png]

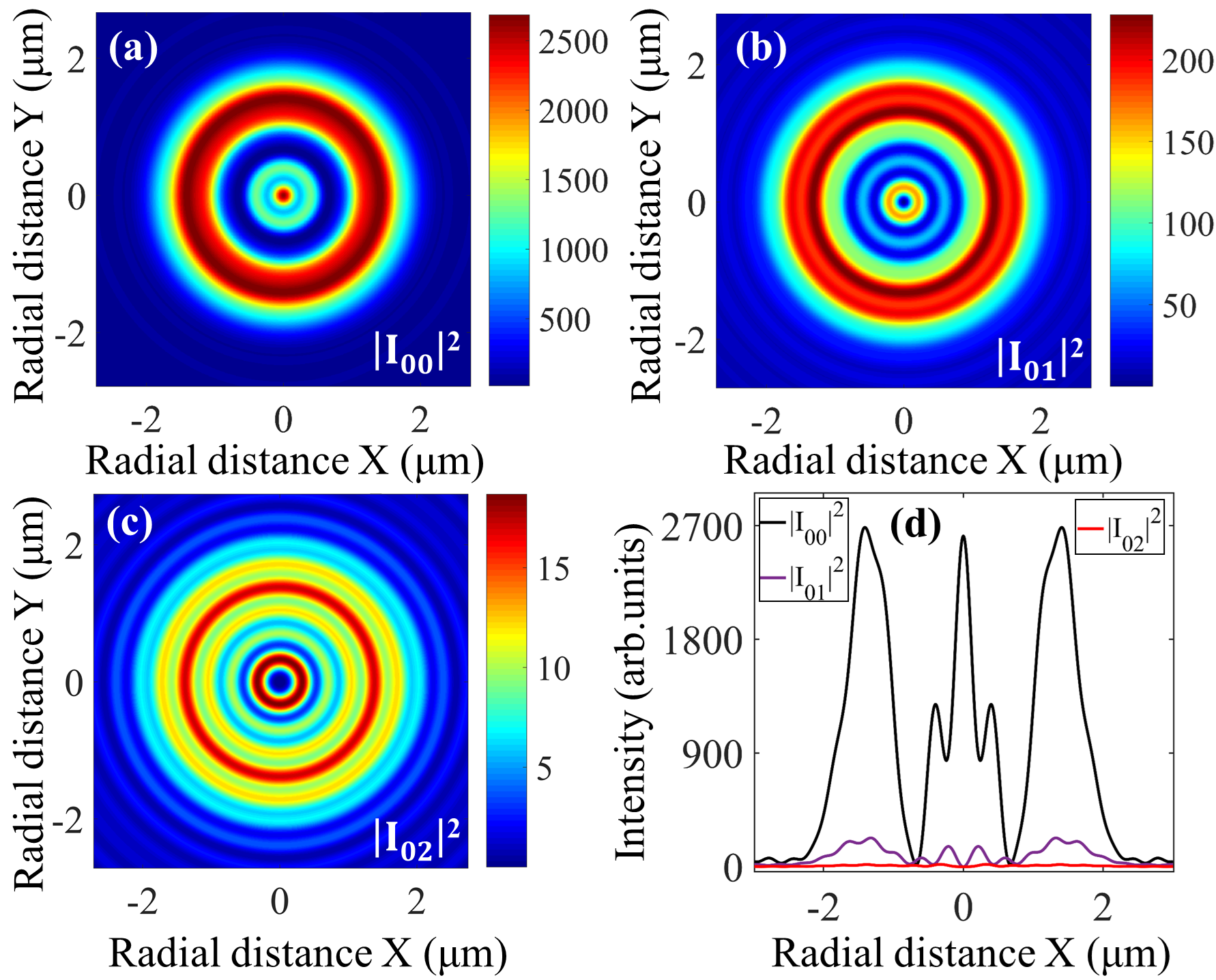

Supplement: Supplementary file 1 — Supporting Information S1 [file NAP2-15-e70034-s001.zip › Fig_1_SM.png]

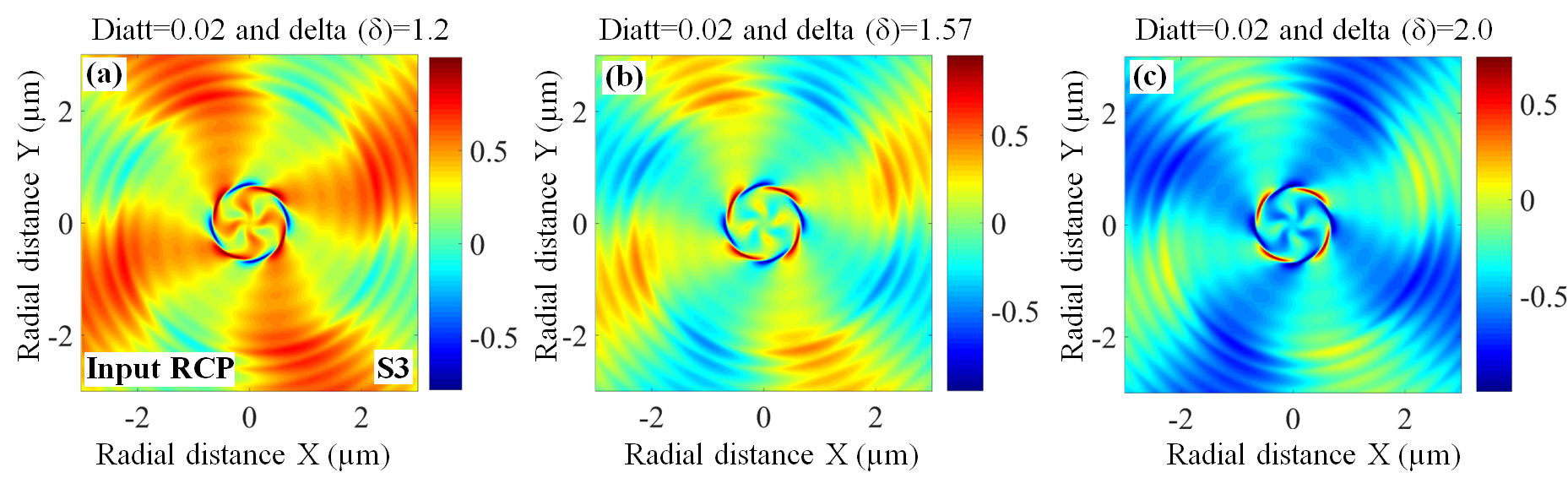

Supplement: Supplementary file 1 — Supporting Information S1 [file NAP2-15-e70034-s001.zip › Fig_6_SM.png]

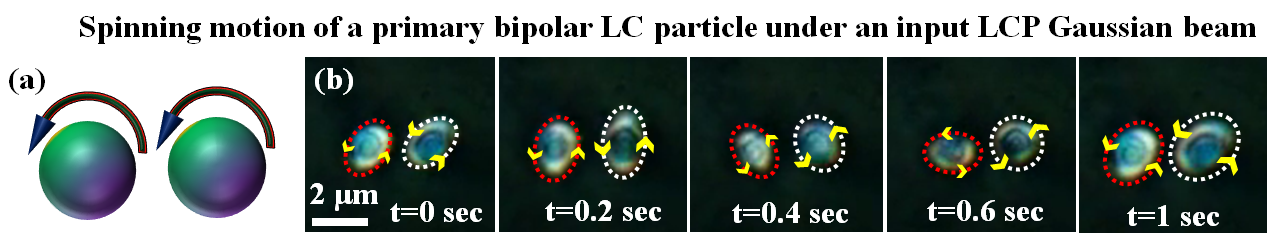

Supplement: Supplementary file 1 — Supporting Information S1 [file NAP2-15-e70034-s001.zip › Fig_7_SM.png]

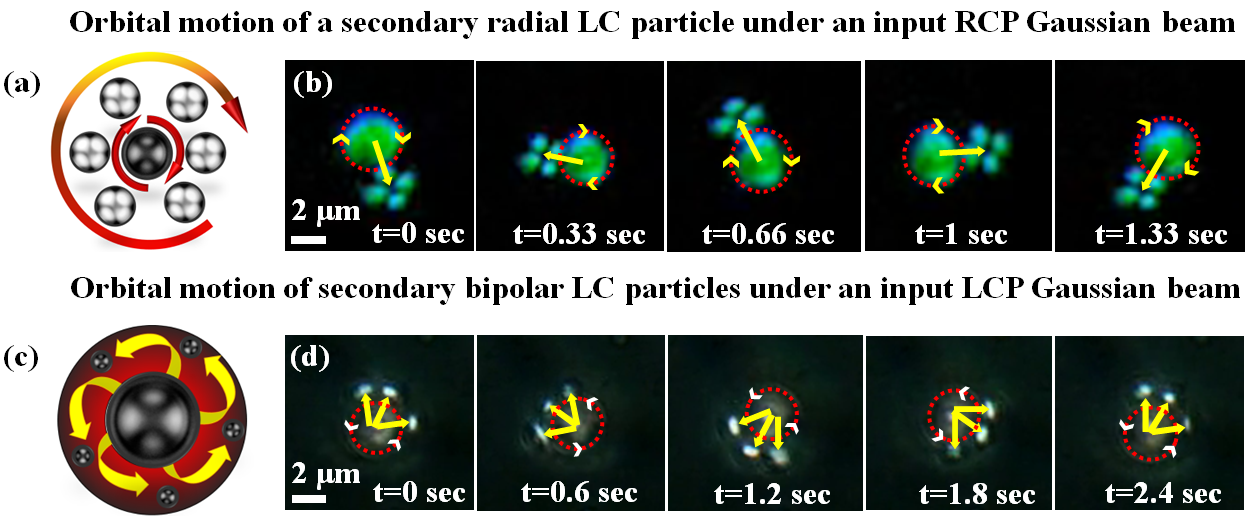

Supplement: Supplementary file 1 — Supporting Information S1 [file NAP2-15-e70034-s001.zip › Fig_8_SM.png]

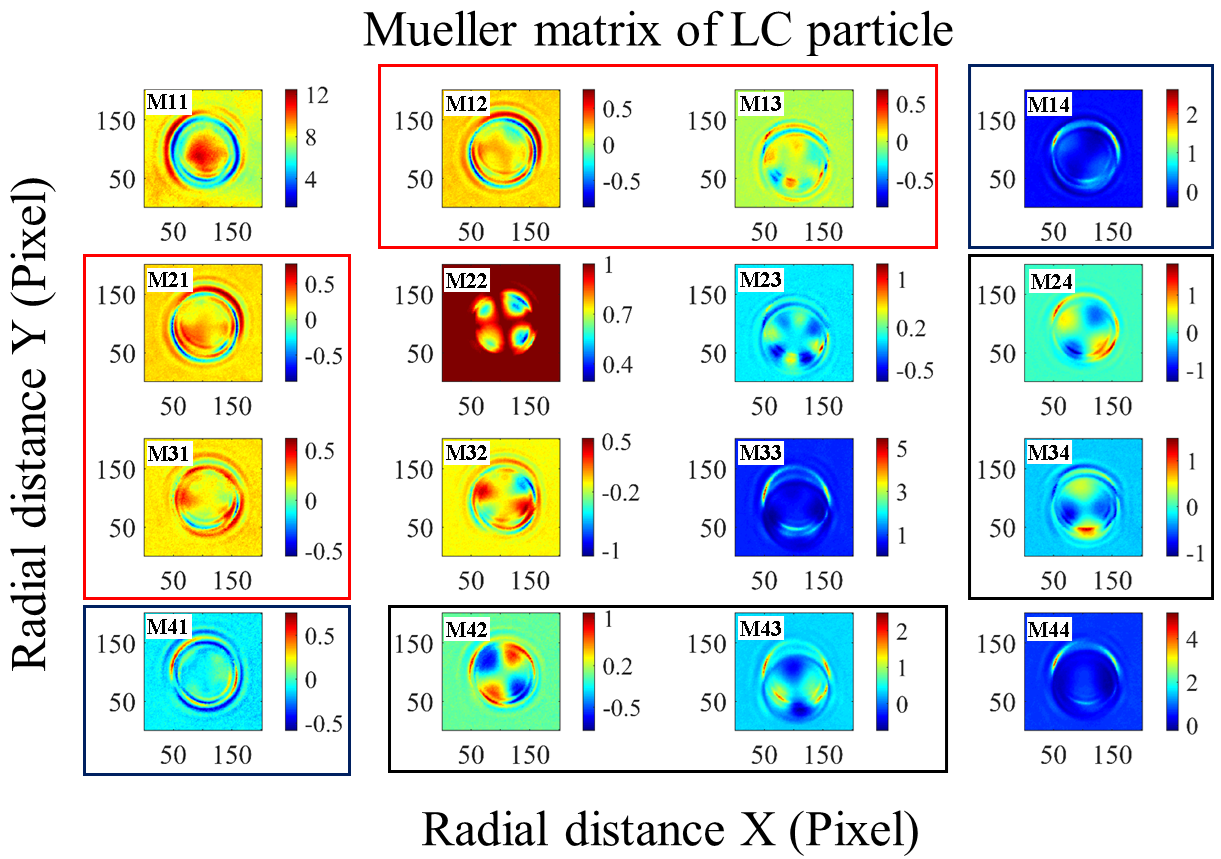

Supplement: Supplementary file 1 — Supporting Information S1 [file NAP2-15-e70034-s001.zip › Fig_9_SM.png]

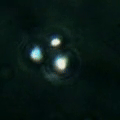

Supplement: Supplementary file 2 — Supporting Information S2 [file NAP2-15-e70034-s002.zip › Videos_gif_files/Video_1_gif.gif]

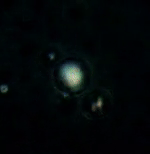

Supplement: Supplementary file 2 — Supporting Information S2 [file NAP2-15-e70034-s002.zip › Videos_gif_files/Video_2_gif.gif]

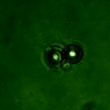

Supplement: Supplementary file 2 — Supporting Information S2 [file NAP2-15-e70034-s002.zip › Videos_gif_files/Video_3_gif.gif]

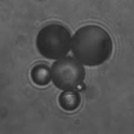

Supplement: Supplementary file 2 — Supporting Information S2 [file NAP2-15-e70034-s002.zip › Videos_gif_files/Video_4_gif.gif]

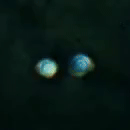

Supplement: Supplementary file 2 — Supporting Information S2 [file NAP2-15-e70034-s002.zip › Videos_gif_files/Video_5_gif.gif]

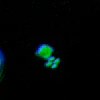

Supplement: Supplementary file 2 — Supporting Information S2 [file NAP2-15-e70034-s002.zip › Videos_gif_files/Video_6_gif.gif]

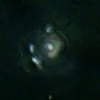

Supplement: Supplementary file 2 — Supporting Information S2 [file NAP2-15-e70034-s002.zip › Videos_gif_files/Video_7_gif.gif]
